# Supplementary figures and images for: Phylogenetic analysis of Fritillaria cirrhosa D. Don and its closely related species based on complete chloroplast genomes
Source: PeerJ. 2019 Aug 21;7:e7480. doi: 10.7717/peerj.7480 (PMC6708372; doi:10.7717/peerj.7480)

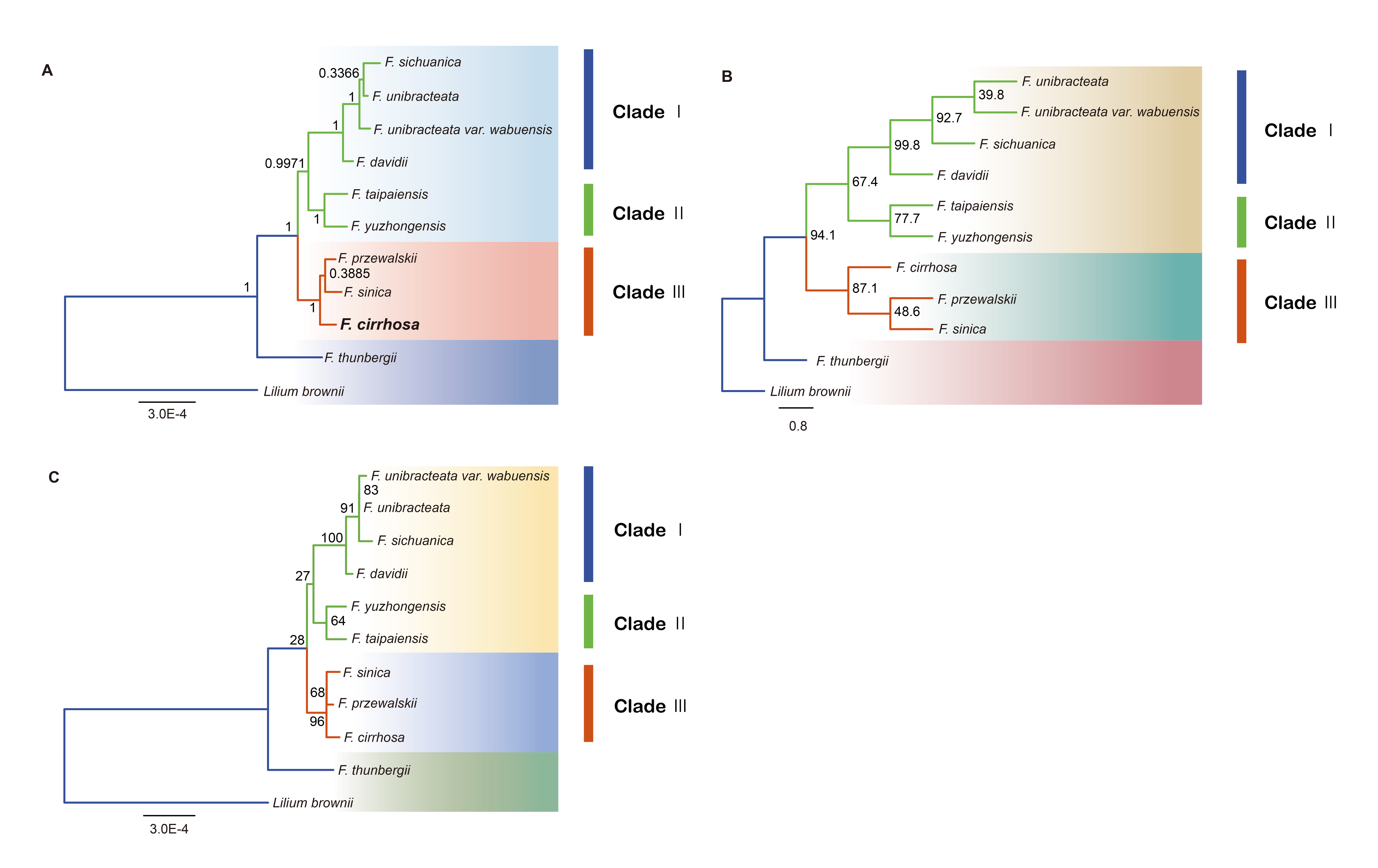

Supplement: Figure S1 — (A) Bayesian analysis (BI), (B) maximum parsimony (MP), and (C) maximum likelihood (ML). [file peerj-07-7480-s001.jpg]
