# Supplementary material for: Phylogenetic analysis of Fritillaria cirrhosa D. Don and its closely related species based on complete chloroplast genomes
Source: PeerJ. 2019 Aug 21;7:e7480. doi: 10.7717/peerj.7480 (PMC6708372; doi:10.7717/peerj.7480)
Supplement: Table S1 [file peerj-07-7480-s003.docx]

Table S1. Some information of nine *Fritillaria* species

|  | Locality | Latitude/Longitude | Altitude(m) | Accession number in Genbank | Voucher specimen |
| --- | --- | --- | --- | --- | --- |
| *F. cirrhosa* | gezan, yunnan, china | N28°08.100 ′/ E099°52.880′ | 4,212 | MH244906 | ZDQ130053 |
| *F. sichuanica* | xiaojin, xichuan, china | N30°42.233′ / E102°22.017′ | 3,935 | MH244907 | ZDQ15022 |
| *F. przewalskii* | ganzi, sichuan, china | N31°33.164′ / E100°00.926′ | 3,682 | MH244908 | ZDQ130018 |
| *F. unibracteata* | hongyuan, sichuan, china | N32°10.532′ / E102°30.686′ | 3,621 | MH244909 | ZDQ13030 |
| *F. taipaiensis* | wuxi, chongqing, china | N31°33.865′ / E109°06.490′ | 2,230 | MH244910 | HCB1 |
| *F. yuzhongensis* | yuzhong, gansu, china | N35°44.158′ / E103°18.873′ | 3,552 | MH244911 | ZDQ14003 |
| *F. sinica* | luding, sichuan, china | N29°32.865′ / E101°58.251′ | 3,900 | MH244912 | ZDQ15023 |
| *F. dajinensis* | jinchuan, sichuan, china | N31°09.676′ / E102°06.704′ | 4,129 | MH244913 | ZDQ15021 |
| *F. thunbergii* | dongyang, zhejiang, china | N29°01.183′ / E120°20.833′ | 320 | MH244914 | ZDQ15009 |
