# Supplementary material for: Phylogenetic analysis of Fritillaria cirrhosa D. Don and its closely related species based on complete chloroplast genomes
Source: PeerJ. 2019 Aug 21;7:e7480. doi: 10.7717/peerj.7480 (PMC6708372; doi:10.7717/peerj.7480)
Supplement: Table S2 [file peerj-07-7480-s004.docx]

Table S2. Amino acid frequencies in protein coding genes of eight *Fritillaria* cp genomes

|  | Ala | Cys | Asp | Glu | Phe | Gly | His | Ile | Lys | Leu | Met | Asn | Pro | Gln | Arg | Ser | Thr | Val | Trp | Tyr |
| --- | --- | --- | --- | --- | --- | --- | --- | --- | --- | --- | --- | --- | --- | --- | --- | --- | --- | --- | --- | --- |
| *F. cirrhosa* | 4.91 | 1.57 | 3.99 | 4.90 | 5.95 | 6.61 | 2.46 | 8.38 | 5.47 | 10.32 | 2.20 | 5.23 | 4.01 | 3.45 | 5.73 | 8.26 | 5.14 | 5.23 | 1.92 | 4.26 |
| *F. sichuanica* | 4.91 | 1.57 | 3.99 | 4.90 | 5.95 | 6.61 | 2.47 | 8.38 | 5.46 | 10.32 | 2.20 | 5.23 | 4.02 | 3.44 | 5.72 | 8.28 | 5.14 | 5.23 | 1.92 | 4.26 |
| *F. przewalskii* | 4.91 | 1.57 | 3.99 | 4.90 | 5.96 | 6.61 | 2.46 | 8.38 | 5.45 | 10.32 | 2.20 | 5.23 | 4.01 | 3.45 | 5.73 | 8.27 | 5.14 | 5.23 | 1.92 | 4.26 |
| *F. unibracteata* | 4.90 | 1.56 | 3.98 | 4.90 | 5.96 | 6.61 | 2.46 | 8.39 | 5.45 | 10.32 | 2.19 | 5.24 | 4.02 | 3.44 | 5.73 | 8.26 | 5.14 | 5.24 | 1.92 | 4.26 |
| *F. taipaiensis* | 4.90 | 1.56 | 3.98 | 4.91 | 5.95 | 6.61 | 2.46 | 8.40 | 5.43 | 10.33 | 2.19 | 5.24 | 4.02 | 3.45 | 5.74 | 8.26 | 5.14 | 5.24 | 1.93 | 4.27 |
| *F. yuzhongensis* | 4.90 | 1.57 | 3.99 | 4.90 | 5.95 | 6.61 | 2.47 | 8.39 | 5.45 | 10.34 | 2.19 | 5.24 | 4.01 | 3.44 | 5.72 | 8.26 | 5.14 | 5.24 | 1.93 | 4.26 |
| *F. sinica* | 4.90 | 1.57 | 3.98 | 4.90 | 5.95 | 6.61 | 2.47 | 8.39 | 5.47 | 10.34 | 2.19 | 5.23 | 4.02 | 3.44 | 5.73 | 8.26 | 5.14 | 5.24 | 1.92 | 4.26 |
| *F. dajinensis* | 4.90 | 1.57 | 3.99 | 4.90 | 5.95 | 6.60 | 2.46 | 8.39 | 5.47 | 10.33 | 2.20 | 5.23 | 4.01 | 3.43 | 5.74 | 8.24 | 5.14 | 5.25 | 1.92 | 4.26 |
