# Supplementary material for: Phylogenetic analysis of Fritillaria cirrhosa D. Don and its closely related species based on complete chloroplast genomes
Source: PeerJ. 2019 Aug 21;7:e7480. doi: 10.7717/peerj.7480 (PMC6708372; doi:10.7717/peerj.7480)
Supplement: Table S3 [file peerj-07-7480-s005.docx]

Table S3. Distribution of simple sequence repeats (SSRs) loci in the eight *Fritillaria* chloroplast genomes.

| NO. | SSR type | SSR | size | start | end | location | locus | region |
| --- | --- | --- | --- | --- | --- | --- | --- | --- |
| *Fritillaria cirrhosa* | | | | | | | | |
| 1 | p1 | (T)12 | 12 | 1,672 | 1,683 | *trnK-UUU* | intron | LSC |
| 2 | p1 | (T)11 | 11 | 1,941 | 1,951 | *matK* | CDS | LSC |
| 3 | p1 | (T)12 | 12 | 4,176 | 4,187 | *trnK-UUU-rps16* | IGS | LSC |
| 4 | p1 | (A)16 | 16 | 6,701 | 6,716 | *psbK-psbI* | IGS | LSC |
| 5 | p2 | (AT)6 | 12 | 7,234 | 7,245 | *trnS-GCU-trnG-GCC* | IGS | LSC |
| 6 | p1 | (T)11 | 11 | 7,648 | 7,658 | *trnS-GCU-trnG-GCC* | IGS | LSC |
| 7 | p1 | (T)18 | 18 | 8,016 | 8,033 | *trnG-GCC* | intron | LSC |
| 8 | p2 | (TA)6 | 12 | 8,786 | 8,797 | *trnR-UCU-atpA* | IGS | LSC |
| 9 | p1 | (T)10 | 10 | 8,798 | 8,807 | *atpA* | CDS | LSC |
| 10 | p1 | (A)13 | 13 | 11,617 | 11,629 | *atpF* | intron | LSC |
| 11 | p1 | (T)10 | 10 | 12,740 | 12,749 | *atpH-atpI* | IGS | LSC |
| 12 | p5 | (TATTG)3 | 15 | 12,928 | 12,942 | *atpH-atpI* | IGS | LSC |
| 13 | p1 | (A)11 | 11 | 12,973 | 12,984 | *atpH-atpI* | IGS | LSC |
| 14 | p3 | (ATA)4 | 12 | 13,026 | 13,037 | *atpH-atpI* | IGS | LSC |
| 15 | p4 | (AATT)3 | 12 | 14,427 | 14,438 | *atpI-rps2* | IGS | LSC |
| 16 | p1 | (T)12 | 12 | 15,197 | 15,208 | *rps2-rpoC2* | IGS | LSC |
| 17 | p1 | (T)12 | 12 | 17,284 | 17,296 | *rpoC2* | CDS | LSC |
| 18 | p1 | (T)11 | 11 | 17,393 | 17,403 | *rpoC2* | CDS | LSC |
| 19 | p2 | (TA)5 | 10 | 18,759 | 18,768 | *rpoC2* | CDS | LSC |
| 20 | p4 | (TAAT)3 | 12 | 27,828 | 27,839 | *psbM-trnD-GUC* | IGS | LSC |
| 21 | p1 | (A)17 | 17 | 28,158 | 28,174 | *psbM-trnD-GUC* | IGS | LSC |
| 22 | p1 | (T)14 | 14 | 28,479 | 28,492 | *trnD-GUC-trnY-GUA* | IGS | LSC |
| 23 | p1 | (A)10 | 10 | 28,513 | 28,522 | *trnY-GUA-trnE-UUC* | IGS | LSC |
| 24 | p2 | (AT)5 | 10 | 29,034 | 29,043 | *trnE-UUC-trnT-GGU* | IGS | LSC |
| 25 | p1 | (T)10 | 10 | 29,211 | 29,220 | *trnE-UUC-trnT-GGU* | IGS | LSC |
| 26 | p1 | (T)18 | 18 | 29,318 | 29,335 | *trnE-UUC-trnT-GGU* | IGS | LSC |
| 27 | p2 | (AT)5 | 10 | 30,007 | 30,016 | *trnT-GGU-psbD* | IGS | LSC |
| 28 | p2 | (AT)5 | 10 | 30,177 | 30,186 | *trnT-GGU-psbD* | IGS | LSC |
| 29 | p1 | (A)10 | 10 | 33,812 | 33,821 | *psbZ-trnG-UCC* | IGS | LSC |
| 30 | p1 | (T)11 | 11 | 39,495 | 39,505 | *psaA-ycf3* | IGS | LSC |
| 31 | p1 | (A)14 | 14 | 39,990 | 40,003 | *psaA-ycf3* | IGS | LSC |
| 32 | p1 | (A)10 | 10 | 41,753 | 41,762 | *ycf3* | intron | LSC |
| 33 | p1 | (A)11 | 11 | 42,787 | 42,797 | *ycf3-trnS-GGA* | IGS | LSC |
| 34 | p1 | (T)12 | 12 | 44,043 | 44,054 | *trnT-UGU-trnL-UAA* | IGS | LSC |
| 35 | p1 | (A)10 | 10 | 44,392 | 44,401 | *trnT-UGU-trnL-UAA* | IGS | LSC |
| 36 | p1 | (A)20 | 20 | 44,587 | 44,606 | *trnT-UGU-trnL-UAA* | IGS | LSC |
| 37 | p2 | (AT)6 | 12 | 45,092 | 45,103 | *trnL-UAA* | intron | LSC |
| 38 | p1 | (T)13 | 13 | 45,406 | 45,418 | *trnL-UAA-trnF-GAA* | IGS | LSC |
| 39 | p2 | (AT)5 | 10 | 45,877 | 45,886 | *trnF-GAA-ndhJ* | IGS | LSC |
| 40 | p1 | (T)12 | 12 | 51,988 | 51,999 | *atpB-rbcL* | IGS | LSC |
| 41 | p1 | (A)11 | 11 | 56,085 | 56,095 | *accD-psaI* | IGS | LSC |
| 42 | p1 | (A)14 | 14 | 56,600 | 56,613 | *accD-psaI* | IGS | LSC |
| 43 | p3 | (GAA)4 | 12 | 56,635 | 56,646 | *accD-psaI* | IGS | LSC |
| 44 | p1 | (A)17 | 17 | 58,618 | 58,634 | *cemA* | CDS | LSC |
| 45 | p2 | (AT)5 | 10 | 61,122 | 61,131 | *petA-psbJ* | IGS | LSC |
| 46 | p1 | (T)13 | 13 | 62,955 | 62,967 | *psbE-petL* | IGS | LSC |
| 47 | p1 | (A)14 | 14 | 63,848 | 63,861 | *trnW-CCA-trnP-UGG* | IGS | LSC |
| 48 | p1 | (A)10 | 10 | 64,306 | 64,315 | *trnP-UGG-psaJ* | IGS | LSC |
| 49 | p1 | (A)10 | 10 | 64,551 | 64,560 | *psaJ-rpl33* | IGS | LSC |
| 50 | p1 | (A)20 | 20 | 64,590 | 64,609 | *psaJ-rpl34* | IGS | LSC |
| 51 | p2 | (AT)5 | 10 | 64,849 | 64,858 | *psaJ-rpl35* | IGS | LSC |
| 52 | p1 | (T)10 | 10 | 68,206 | 68,215 | *clpP* | intron | LSC |
| 53 | p1 | (A)11 | 11 | 68593 | 68603 | *clpP* | intron | LSC |
| 54 | p1 | (T)12 | 12 | 68,916 | 68,927 | *clpP* | intron | LSC |
| 55 | p1 | (A)18 | 18 | 72,490 | 72,507 | *petB* | intron | LSC |
| 56 | p2 | (AT)7 | 14 | 75,391 | 75,404 | *petD-rpoA* | IGS | LSC |
| 57 | p4 | (TTCT)3 | 12 | 79,859 | 79,870 | *rpl16* | intron | LSC |
| 58 | p1 | (T)17 | 17 | 80,247 | 80,263 | *rps3* | CDS | LSC |
| 59 | p1 | (A)20 | 20 | 81,485 | 81,504 | *rpl22-rps19* | IGS | LSC |
| 60 | p1 | (T)10 | 10 | 82,938 | 82,947 | *rpl2* | intron | IRb |
| 61 | p2 | (GA)5 | 10 | 87,215 | 87,224 | *ycf2* | CDS | IRb |
| 62 | p1 | (A)10 | 10 | 110,417 | 110,426 | *ndhF-rpl32* | IGS | SSC |
| 63 | p1 | (T)14 | 14 | 111,301 | 111,314 | *rpl32-trnL-UAG* | IGS | SSC |
| 64 | p1 | (A)13 | 13 | 111,359 | 111,371 | *rpl32-trnL-UAG* | IGS | SSC |
| 65 | p4 | (AATA)3 | 12 | 113,140 | 113,151 | *ndhD* | CDS | SSC |
| 66 | p1 | (A)10 | 10 | 115,910 | 115,919 | *ndhG* | CDS | SSC |
| 67 | p4 | (AAAT)4 | 16 | 116,255 | 116,270 | *ndhG-ndhI* | IGS | SSC |
| 68 | p1 | (A)11 | 11 | 116,568 | 116,578 | *ndhG-ndhI* | IGS | SSC |
| 69 | p2 | (TC)5 | 10 | 120,152 | 120,161 | *ndhH* | CDS | SSC |
| 70 | p1 | (T)14 | 14 | 121,167 | 121,187 | *rps15-ycf1* | IGS | SSC |
| 71 | p4 | (AAAT)3 | 12 | 121,221 | 121,232 | *rps15-ycf1* | IGS | SSC |
| 72 | p1 | (T)10 | 10 | 121,667 | 121,676 | *ycf1* | CDS | SSC |
| 73 | p1 | (T)15 | 15 | 122,282 | 122,296 | *ycf1* | CDS | SSC |
| 74 | p1 | (T)14 | 14 | 122,495 | 122,508 | *ycf1* | CDS | SSC |
| 75 | p1 | (T)16 | 16 | 122,944 | 122,959 | *ycf1* | CDS | SSC |
| 76 | p1 | (T)11 | 11 | 123,504 | 123,514 | *ycf1* | CDS | SSC |
| 77 | p2 | (TC)5 | 10 | 146,530 | 146,539 | *ycf2* | CDS | IRa |
| 78 | p1 | (A)10 | 10 | 150,807 | 150,816 | *rpl2* | intron | IRa |
| *Fritillaria sichuanica* | | | | | | | | |
| 1 | p1 | (T)17 | 17 | 1,774 | 1,790 | *trnK-UUU* | intron | LSC |
| 2 | p1 | (T)10 | 10 | 3,693 | 3,702 | *trnK-UUU* | intron | LSC |
| 3 | p1 | (A)14 | 14 | 4,107 | 4,120 | *trnK-UUU-rps16* | IGS | LSC |
| 4 | p1 | (A)10 | 10 | 4,137 | 4,146 | *trnK-UUU-rps16* | IGS | LSC |
| 5 | p1 | (T)13 | 13 | 4,315 | 4,327 | *trnK-UUU-rps16* | IGS | LSC |
| 6 | p1 | (T)10 | 10 | 6,173 | 6,182 | *rps16-trnQ-UUG* | IGS | LSC |
| 7 | p1 | (A)19 | 19 | 6,845 | 6,863 | *psbK-psbI* | IGS | LSC |
| 8 | p2 | (AT)6 | 12 | 7,379 | 7,390 | *trnS-GCU-trnG-GCC* | IGS | LSC |
| 9 | p1 | (T)14 | 14 | 7,808 | 7,821 | *trnS-GCU-trnG-GCC* | IGS | LSC |
| 10 | p4 | (AGAA)3 | 12 | 8,823 | 8,834 | *trnG-GCC* | intron | LSC |
| 11 | p2 | (TA)6 | 12 | 8,989 | 9,000 | *trnR-UCU-atpA* | IGS | LSC |
| 12 | p1 | (A)12 | 12 | 11,818 | 11,829 | *atpF* | intron | LSC |
| 13 | p1 | (A)12 | 12 | 13,167 | 13,178 | *atpH-atpI* | IGS | LSC |
| 14 | p4 | (AATT)3 | 12 | 14,624 | 14,635 | *atpI-rps2* | IGS | LSC |
| 15 | p1 | (T)15 | 15 | 15,394 | 15,408 | *rps2-rpoC2* | IGS | LSC |
| 16 | p1 | (T)12 | 12 | 17,484 | 17,496 | *rpoC2* | CDS | LSC |
| 17 | p1 | (T)11 | 11 | 17,593 | 17,603 | *rpoC2* | CDS | LSC |
| 18 | p2 | (TA)5 | 10 | 18,959 | 18,968 | *rpoC2* | CDS | LSC |
| 19 | p4 | (TAAT)3 | 12 | 28,008 | 28,019 | *psbM-trnD-GUC* | IGS | LSC |
| 20 | p1 | (A)18 | 18 | 28,342 | 28,359 | *psbM-trnD-GUC* | IGS | LSC |
| 21 | p1 | (T)14 | 14 | 28,664 | 28,678 | *trnD-GUC-trnY-GUA* | IGS | LSC |
| 22 | p1 | (A)16 | 16 | 28,697 | 28,712 | *trnD-GUC-trnY-GUA* | IGS | LSC |
| 23 | p2 | (AT)5 | 10 | 29,220 | 29,229 | *trnE-UUC-trnT-GGU* | IGS | LSC |
| 24 | p1 | (T)11 | 11 | 29,392 | 29,403 | *trnE-UUC-trnT-GGU* | IGS | LSC |
| 25 | p1 | (T)18 | 18 | 29,500 | 29,517 | *trnE-UUC-trnT-GGU* | IGS | LSC |
| 26 | p5 | (TATTA)3 | 15 | 29,626 | 29,640 | *trnE-UUC-trnT-GGU* | IGS | LSC |
| 27 | p1 | (A)12 | 12 | 29,832 | 29,843 | *trnT-GGU-psbD* | IGS | LSC |
| 28 | p2 | (AT)5 | 10 | 30,200 | 30,209 | *trnT-GGU-psbD* | IGS | LSC |
| 29 | p2 | (AT)5 | 10 | 30,388 | 30,397 | *trnT-GGU-psbD* | IGS | LSC |
| 30 | p1 | (A)11 | 11 | 34,022 | 34,032 | *psbZ-trnG-UCC* | IGS | LSC |
| 31 | p1 | (T)10 | 10 | 39,708 | 39,717 | *psaA-ycf3* | IGS | LSC |
| 32 | p1 | (A)12 | 12 | 40,201 | 40,212 | *psaA-ycf3* | IGS | LSC |
| 33 | p1 | (A)12 | 12 | 41,961 | 41,973 | *ycf3* | intron | LSC |
| 34 | p2 | (TC)5 | 10 | 42,008 | 42,017 | *ycf3* | intron | LSC |
| 35 | p1 | (T)12 | 12 | 44,211 | 44,222 | *trnT-UGU-trnL-UAA* | IGS | LSC |
| 36 | p1 | (A)14 | 14 | 44,580 | 44,593 | *trnT-UGU-trnL-UAA* | IGS | LSC |
| 37 | p1 | (A)19 | 19 | 44,777 | 44,795 | *trnT-UGU-trnL-UAA* | IGS | LSC |
| 38 | p2 | (AT)6 | 12 | 45,281 | 45,292 | *trnL-UAA* | intron | LSC |
| 39 | p1 | (T)11 | 11 | 45,595 | 45,605 | *trnL-UAA-trnF-GAA* | IGS | LSC |
| 40 | p1 | (A)17 | 17 | 56,264 | 56,280 | *accD-psaI* | IGS | LSC |
| 41 | p1 | (A)13 | 13 | 56,793 | 56,806 | *accD-psaI* | IGS | LSC |
| 42 | p3 | (GAA)4 | 12 | 56,827 | 56,838 | *accD-psaI* | IGS | LSC |
| 43 | p1 | (A)14 | 14 | 58,767 | 58,780 | *cemA* | CDS | LSC |
| 44 | p2 | (AT)5 | 10 | 61,272 | 61,281 | *petA-psbJ* | IGS | LSC |
| 45 | p1 | (A)20 | 20 | 62,977 | 62,996 | *petA-psbJ* | IGS | LSC |
| 46 | p1 | (T)15 | 15 | 63,103 | 63,117 | *petA-psbJ* | IGS | LSC |
| 47 | p1 | (A)15 | 15 | 63,998 | 64,012 | *trnW-CCA-trnP-UGG* | IGS | LSC |
| 48 | p1 | (A)14 | 14 | 64,706 | 64,719 | *psaJ-rpl33* | IGS | LSC |
| 49 | p1 | (T)13 | 13 | 68,312 | 68,324 | *clpP* | intron | LSC |
| 50 | p1 | (A)14 | 14 | 68,702 | 68,715 | *clpP* | intron | LSC |
| 51 | p1 | (T)13 | 13 | 69,028 | 69,040 | *clpP* | intron | LSC |
| 52 | p1 | (A)11 | 11 | 72,603 | 72,613 | *petB* | intron | LSC |
| 53 | p2 | (AT)7 | 14 | 75,476 | 75,489 | *petD-rpoA* | IGS | LSC |
| 54 | p3 | (TTCT)3 | 12 | 79,945 | 79,956 | *rpl16* | intron | LSC |
| 55 | p1 | (T)11 | 11 | 80,018 | 80,028 | *rpl16* | intron | LSC |
| 56 | p1 | (T)12 | 12 | 80,323 | 80,334 | *rpl16-rps3* | IGS | LSC |
| 57 | p2 | (GA)5 | 10 | 87,274 | 87,283 | *ycf2* | CDS | IRb |
| 58 | p1 | (A)10 | 10 | 110,479 | 110,488 | *ndhF-rpl32* | IGS | SSC |
| 59 | p1 | (T)19 | 19 | 111,363 | 111,381 | *rpl32-trnL-UAG* | IGS | SSC |
| 60 | p1 | (A)21 | 21 | 111,426 | 111,446 | *rpl33-trnL-UAG* | IGS | SSC |
| 61 | p4 | (AATA)3 | 12 | 113,212 | 113,223 | *ndhD* | CDS | SSC |
| 62 | p1 | (A)10 | 10 | 115,982 | 115,991 | *ndhG* | CDS | SSC |
| 63 | p4 | (AAAT)4 | 16 | 116,327 | 116,342 | *ndhG-ndhI* | IGS | SSC |
| 64 | p1 | (A)11 | 11 | 116,640 | 116,650 | *ndhG-ndhI* | IGS | SSC |
| 65 | p2 | (TC)5 | 10 | 120,222 | 120,231 | *ndhH* | CDS | SSC |
| 66 | p1 | (T)14 | 14 | 121,237 | 121,250 | *rps15-ycf1* | IGS | SSC |
| 67 | p4 | (AAAT)3 | 12 | 121,292 | 121,303 | *rps15-ycf2* | IGS | SSC |
| 68 | p1 | (T)10 | 10 | 121,738 | 121,747 | *ycf1* | CDS | SSC |
| 69 | p1 | (T)15 | 15 | 122,353 | 122,367 | *ycf1* | CDS | SSC |
| 70 | p1 | (T)14 | 14 | 122,566 | 122,579 | *ycf1* | CDS | SSC |
| 71 | p1 | (T)10 | 10 | 123,009 | 123,018 | *ycf1* | CDS | SSC |
| 72 | p1 | (T)11 | 11 | 123,563 | 123,573 | *ycf1* | CDS | SSC |
| 73 | p2 | (TC)5 | 10 | 146,592 | 146,601 | *ycf2* | CDS | IRa |
| *Fritillaria przewalskii* | | | | | | | | |
| 1 | p1 | (T)13 | 13 | 1,774 | 1,786 | *trnK-UUU* | intron | LSC |
| 2 | p1 | (T)11 | 11 | 2,044 | 2,054 | *matK* | CDS | LSC |
| 3 | p1 | (A)14 | 14 | 4,103 | 4,116 | *trnK-UUU* | intron | LSC |
| 4 | p1 | (T)15 | 15 | 4,285 | 4,299 | *trnK-UUU-rps16* | IGS | LSC |
| 5 | p2 | (TA)6 | 12 | 5,612 | 5,623 | *rps16* | intron | LSC |
| 6 | p1 | (A)19 | 19 | 6,814 | 6,832 | *psbK-psbI* | IGS | LSC |
| 7 | p2 | (AT)6 | 12 | 7,348 | 7,359 | *trnS-GCU-trnG-GCC* | IGS | LSC |
| 8 | p1 | (T)12 | 12 | 7,776 | 7,787 | *trnS-GCU-trnG-GCC* | IGS | LSC |
| 9 | p1 | (T)15 | 15 | 8,145 | 8,159 | *trnG-GCC* | intron | LSC |
| 10 | p2 | (TA)6 | 12 | 8,913 | 8,924 | *trnR-UCU-atpA* | IGS | LSC |
| 11 | p1 | (T)10 | 10 | 8,925 | 8,934 | *trnR-UCU-atpA* | IGS | LSC |
| 12 | p1 | (A)15 | 15 | 11,743 | 11,757 | *atpF* | intron | LSC |
| 13 | p1 | (T)10 | 10 | 12,869 | 12,878 | *atpH-atpI* | IGS | LSC |
| 14 | p1 | (A)11 | 11 | 13,097 | 13,108 | *atpH-atpI* | IGS | LSC |
| 15 | p3 | (ATA)4 | 12 | 13,150 | 13,161 | *atpH-atpI* | IGS | LSC |
| 16 | p4 | (AATT)3 | 12 | 14,550 | 14,561 | *atpI-rps2* | IGS | LSC |
| 17 | p1 | (T)13 | 13 | 15,320 | 15,332 | *rps2-rpoC2* | IGS | LSC |
| 18 | p1 | (T)12 | 12 | 17,408 | 17,419 | *rpoC2* | CDS | LSC |
| 19 | p1 | (T)11 | 11 | 17,517 | 17,527 | *rpoC2* | CDS | LSC |
| 20 | p2 | (TA)5 | 10 | 18,883 | 18,892 | *rpoC2* | CDS | LSC |
| 21 | p4 | (TAAT)3 | 12 | 27,953 | 27,964 | *psbM-trnD-GUC* | IGS | LSC |
| 22 | p1 | (A)20 | 20 | 28,283 | 28,302 | *psbM-trnD-GUC* | IGS | LSC |
| 23 | p1 | (T)15 | 15 | 28,607 | 28,622 | *trnD-GUC-trnY-GUA* | IGS | LSC |
| 24 | p1 | (A)18 | 18 | 28,642 | 28,659 | *trnD-GUC-trnY-GUA* | IGS | LSC |
| 25 | p2 | (AT)5 | 10 | 29,170 | 29,179 | *trnE-UUC-trnT-GGU* | IGS | LSC |
| 26 | p1 | (T)18 | 18 | 29,453 | 29,470 | *trnE-UUC-trnT-GGU* | IGS | LSC |
| 27 | p1 | (A)11 | 11 | 29,780 | 29,790 | *trnT-GGU-psbD* | IGS | LSC |
| 28 | p2 | (AT)5 | 10 | 30,144 | 30,153 | *trnT-GGU-psbD* | IGS | LSC |
| 29 | p2 | (AT)5 | 10 | 30,314 | 30,323 | *trnT-GGU-psbD* | IGS | LSC |
| 30 | p1 | (A)11 | 11 | 33,955 | 33,965 | *psbZ-trnG-UCC* | IGS | LSC |
| 31 | p1 | (T)11 | 11 | 39,639 | 39,649 | *psaA-ycf3* | IGS | LSC |
| 32 | p1 | (A)15 | 15 | 40,134 | 40,148 | *psaA-ycf3* | IGS | LSC |
| 33 | p1 | (A)10 | 10 | 41,898 | 41,907 | *ycf3* | intron | LSC |
| 34 | p1 | (A)10 | 10 | 42,932 | 42,941 | *trnS-GGA-rps4* | IGS | LSC |
| 35 | p1 | (A)12 | 12 | 42,977 | 42,988 | *trnS-GGA-rps5* | IGS | LSC |
| 36 | p1 | (T)11 | 11 | 44,190 | 44,200 | *trnT-UGU-trnL-UAA* | IGS | LSC |
| 37 | p1 | (A)15 | 15 | 44,538 | 44,552 | *trnT-UGU-trnL-UAA* | IGS | LSC |
| 38 | p1 | (A)19 | 19 | 44,738 | 44,756 | *trnT-UGU-trnL-UAA* | IGS | LSC |
| 39 | p2 | (AT)6 | 12 | 45,242 | 45,253 | *trnL-UAA* | intron | LSC |
| 40 | p1 | (T)16 | 16 | 45,556 | 45,571 | *trnL-UAA-trnF-GAA* | IGS | LSC |
| 41 | p1 | (T)11 | 11 | 52,136 | 52,146 | *atpB-rbcL* | IGS | LSC |
| 42 | p1 | (A)11 | 11 | 56,233 | 56,243 | *accD-psaI* | IGS | LSC |
| 43 | p1 | (A)16 | 16 | 56,748 | 56,763 | *accD-psaI* | IGS | LSC |
| 44 | p3 | (GAA)4 | 12 | 56,785 | 56,796 | *accD-psaI* | IGS | LSC |
| 45 | p1 | (A)13 | 13 | 58,768 | 58,780 | *cemA* | CDS | LSC |
| 46 | p1 | (T)16 | 16 | 63,101 | 63,116 | *psbE-petL* | IGS | LSC |
| 47 | p1 | (A)14 | 14 | 63,997 | 64,010 | *trnW-CCA-trnP-UGG* | IGS | LSC |
| 48 | p1 | (A)20 | 20 | 64,699 | 64,718 | *psaJ-rpl33* | IGS | LSC |
| 49 | p2 | (AT)6 | 12 | 64,958 | 64,969 | *psaJ-rpl33* | IGS | LSC |
| 50 | p1 | (T)10 | 10 | 68,312 | 68,321 | *clpP* | intron | LSC |
| 51 | p1 | (A)12 | 12 | 68,699 | 68710 | *clpP* | intron | LSC |
| 52 | p1 | (A)19 | 19 | 72,595 | 72,613 | *petB* | intron | LSC |
| 53 | p2 | (AT)7 | 14 | 75,497 | 75,510 | *petD-rpoA* | IGS | LSC |
| 54 | p4 | (TTCT)3 | 12 | 79,965 | 79,976 | *rpl16* | intron | LSC |
| 55 | p1 | (T)20 | 20 | 80,352 | 80,371 | *rpl16-rps3* | IGS | LSC |
| 56 | p1 | (A)16 | 16 | 81,573 | 81,588 | *rpl22-rps19* | IGS | LSC |
| 57 | p1 | (T)10 | 10 | 83,022 | 83,031 | *rpl2* | intron | IRb |
| 58 | p2 | (GA)5 | 10 | 87,299 | 87,308 | *ycf2* | CDS | IRb |
| 59 | p1 | (A)10 | 10 | 110,502 | 110,511 | *ndhF-rpl32* | IGS | SSC |
| 60 | p1 | (T)12 | 12 | 111,386 | 111,397 | *rpl32-trnL-UAG* | IGS | SSC |
| 61 | p1 | (A)16 | 16 | 111,442 | 111,457 | *rpl32-trnL-UAG* | IGS | SSC |
| 62 | p4 | (AATA)3 | 12 | 113,226 | 113,237 | *ndhD* | CDS | SSC |
| 63 | p1 | (A)10 | 10 | 115,996 | 116,005 | *psaC-ndhE* | IGS | SSC |
| 64 | p4 | (AAAT)4 | 16 | 116,341 | 116,356 | *ndhG-ndhI* | IGS | SSC |
| 65 | p2 | (TC)5 | 10 | 120,236 | 120,245 | *ndhH* | CDS | SSC |
| 66 | p1 | (T)15 | 15 | 121,251 | 121,265 | *rps15-ycf1* | IGS | SSC |
| 67 | p4 | (AAAT)3 | 12 | 121,306 | 121,317 | *rps15-ycf1* | IGS | SSC |
| 68 | p1 | (T)10 | 10 | 121,752 | 121,761 | *ycf1* | CDS | SSC |
| 69 | p1 | (T)15 | 15 | 122,367 | 122,381 | *ycf1* | CDS | SSC |
| 70 | p1 | (T)14 | 14 | 122,580 | 122,593 | *ycf1* | CDS | SSC |
| 71 | p1 | (T)16 | 16 | 123,023 | 123,038 | *ycf1* | CDS | SSC |
| 72 | p1 | (T)11 | 11 | 123,583 | 123,593 | *ycf1* | CDS | SSC |
| 73 | p2 | (TC)5 | 10 | 146,610 | 146,619 | *ycf2* | CDS | IRa |
| 74 | p1 | (A)10 | 10 | 150,887 | 150,896 | *rpl2* | intron | IRa |
| *Fritillaria unibracteata* | | | | | | | | |
| 1 | p1 | (T)17 | 17 | 1,679 | 1,695 | *trnK-UUU* | intron | LSC |
| 2 | p1 | (T)10 | 10 | 3,598 | 3,607 | *trnK-UUU* | intron | LSC |
| 3 | p1 | (A)19 | 19 | 4,012 | 4,030 | *trnK-UUU-rps16* | IGS | LSC |
| 4 | p1 | (T)13 | 13 | 4,224 | 4,236 | *trnK-UUU-rps16* | IGS | LSC |
| 5 | p1 | (T)10 | 10 | 4,703 | 4,712 | *trnK-UUU-rps16* | IGS | LSC |
| 6 | p1 | (C)10 | 10 | 4,970 | 4,979 | *rps16* | intron | LSC |
| 7 | p1 | (T)12 | 12 | 6,080 | 6,091 | *rps16-trnQ-UUG* | IGS | LSC |
| 8 | p1 | (A)18 | 18 | 6,754 | 6,771 | *psbK-psbI* | IGS | LSC |
| 9 | p2 | (AT)6 | 12 | 7,287 | 7,298 | *trnS-GCU-trnG-GCC* | IGS | LSC |
| 10 | p1 | (T)19 | 19 | 7,730 | 7,748 | *trnS-GCU-trnG-GCC* | IGS | LSC |
| 11 | p1 | (T)10 | 10 | 8,106 | 8,115 | *trnG-GCC* | intron | LSC |
| 12 | p4 | (AGAA)3 | 12 | 8,751 | 8,762 | *trnG-GCC-trnR-UCU* | IGS | LSC |
| 13 | p2 | (TA)6 | 12 | 8,870 | 8,881 | *trnR-UCU-atpA* | IGS | LSC |
| 14 | p1 | (A)11 | 11 | 11,700 | 11,710 | *atpF* | intron | LSC |
| 15 | p1 | (A)11 | 11 | 13,049 | 13,059 | *atpH-atpI* | IGS | LSC |
| 16 | p4 | (AATT)3 | 12 | 14,505 | 14,516 | *atpI-rps2* | IGS | LSC |
| 17 | p1 | (T)16 | 16 | 15,275 | 15,290 | *rps2-rpoC2* | IGS | LSC |
| 18 | p1 | (T)12 | 12 | 17,366 | 17,377 | *rpoC2* | CDS | LSC |
| 19 | p1 | (T)11 | 11 | 17,475 | 17,485 | *rpoC2* | CDS | LSC |
| 20 | p2 | (TA)5 | 10 | 18,841 | 18,850 | *rpoC2* | CDS | LSC |
| 21 | p4 | (TAAT)3 | 12 | 27,879 | 27,890 | *psbM-trnD-GUC* | IGS | LSC |
| 22 | p1 | (A)22 | 22 | 28,213 | 28,234 | *psbM-trnD-GUC* | IGS | LSC |
| 23 | p1 | (T)17 | 17 | 28,539 | 28,555 | *trnD-GUC-trnY-GUA* | IGS | LSC |
| 24 | p1 | (A)11 | 11 | 28,576 | 28,586 | *trnD-GUC-trnY-GUA* | IGS | LSC |
| 25 | p2 | (AT)5 | 10 | 28,586 | 28,595 | *trnD-GUC-trnY-GUA* | IGS | LSC |
| 26 | p2 | (AT)5 | 10 | 29,103 | 29,112 | *trnE-UUC-trnT-GGU* | IGS | LSC |
| 27 | p1 | (T)16 | 16 | 29,380 | 29,395 | *trnE-UUC-trnT-GGU* | IGS | LSC |
| 28 | p1 | (A)18 | 18 | 29,705 | 29,722 | *trnT-GGU-psbD* | IGS | LSC |
| 29 | p2 | (AT)5 | 10 | 30,077 | 30,086 | *trnT-GGU-psbD* | IGS | LSC |
| 30 | p2 | (AT)5 | 10 | 30,256 | 30,265 | *trnT-GGU-psbD* | IGS | LSC |
| 31 | p1 | (A)11 | 11 | 33,890 | 33,900 | *psbZ-trnG-UCC* | IGS | LSC |
| 32 | p1 | (T)11 | 11 | 39,576 | 39,586 | *psaA-ycf3* | IGS | LSC |
| 33 | p1 | (A)15 | 15 | 40,071 | 40,085 | *psaA-ycf3* | IGS | LSC |
| 34 | p1 | (A)17 | 17 | 41,834 | 41,850 | *ycf3* | intron | LSC |
| 35 | p2 | (TC)5 | 10 | 41,885 | 41,894 | *ycf3* | intron | LSC |
| 36 | p1 | (A)11 | 11 | 42,876 | 42,886 | *trnS-GGA-rps4* | IGS | LSC |
| 37 | p1 | (T)15 | 15 | 44,089 | 44,103 | *trnT-UGU-trnL-UAA* | IGS | LSC |
| 38 | p1 | (A)19 | 19 | 44,462 | 44,480 | *trnT-UGU-trnL-UAA* | IGS | LSC |
| 39 | p1 | (A)20 | 20 | 44,663 | 44,682 | *trnT-UGU-trnL-UAA* | IGS | LSC |
| 40 | p2 | (AT)6 | 12 | 45,168 | 45,179 | *trnL-UAA* | intron | LSC |
| 41 | p1 | (T)12 | 12 | 45,482 | 45,493 | *trnL-UAA-trnF-GAA* | IGS | LSC |
| 42 | p1 | (A)15 | 15 | 56,146 | 56,160 | *accD-psaI* | IGS | LSC |
| 43 | p1 | (A)13 | 13 | 56,673 | 56,685 | *accD-psaI* | IGS | LSC |
| 44 | p3 | (GAA)4 | 12 | 56,707 | 56,718 | *accD-psaI* | IGS | LSC |
| 45 | p1 | (A)20 | 20 | 58,219 | 58,238 | *cemA* | CDS | LSC |
| 46 | p2 | (AT)5 | 10 | 60,730 | 60,739 | *petA-psbJ* | IGS | LSC |
| 47 | p1 | (A)18 | 18 | 62,435 | 62,452 | *psbE-petL* | IGS | LSC |
| 48 | p1 | (T)13 | 13 | 62,559 | 62,571 | *psbE-petL* | IGS | LSC |
| 49 | p1 | (A)16 | 16 | 63,452 | 63,467 | *trnW-CCA-trnP-UGG* | IGS | LSC |
| 50 | p1 | (A)14 | 14 | 64,161 | 64,174 | *psaJ-rpl33* | IGS | LSC |
| 51 | p1 | (A)19 | 19 | 64,204 | 64,222 | *psaJ-rpl33* | IGS | LSC |
| 52 | p1 | (T)16 | 16 | 67815 | 67,830 | *clpP* | intron | LSC |
| 53 | p1 | (A)11 | 11 | 68,208 | 68,218 | *clpP* | intron | LSC |
| 54 | p1 | (T)11 | 11 | 68,531 | 68,541 | *clpP* | intron | LSC |
| 55 | p1 | (A)11 | 11 | 72,104 | 72,114 | *petB* | intron | LSC |
| 56 | p2 | (AT)7 | 14 | 74,987 | 75,000 | *petD-rpoA* | IGS | LSC |
| 57 | p4 | (TTCT)3 | 12 | 79,456 | 79,467 | *rpl16* | intron | LSC |
| 58 | p1 | (T)10 | 10 | 79,529 | 79,538 | *rpl16* | intron | LSC |
| 59 | p1 | (T)16 | 16 | 79,837 | 79,852 | *rpl16-rps3* | IGS | LSC |
| 60 | p1 | (A)14 | 14 | 81,075 | 81,088 | *rpl22-rps19* | IGS | LSC |
| 61 | p2 | (GA)5 | 10 | 86,798 | 86,807 | *ycf2* | CDS | IRb |
| 62 | p1 | (A)10 | 10 | 109,742 | 109,751 | *ndhF-rpl32* | IGS | SSC |
| 63 | p1 | (T)11 | 11 | 110,626 | 110,636 | *rpl32-trnL-UAG* | IGS | SSC |
| 64 | p1 | (A)20 | 20 | 110,681 | 110,700 | *rpl33-trnL-UAG* | IGS | SSC |
| 65 | p4 | (AATA)3 | 12 | 112,466 | 112,477 | *ndhD* | CDS | SSC |
| 66 | p1 | (A)10 | 10 | 115,236 | 115,245 | *ndhG* | CDS | SSC |
| 67 | p4 | (AAAT)4 | 16 | 115,581 | 115,596 | *ndhG-ndhI* | IGS | SSC |
| 68 | p1 | (A)11 | 11 | 115,894 | 115,904 | *ndhG-ndhI* | IGS | SSC |
| 69 | p2 | (TC)5 | 10 | 119476 | 119485 | *ndhH* | CDS | SSC |
| 70 | p1 | (T)14 | 14 | 120,491 | 120,504 | *rps15-ycf1* | IGS | SSC |
| 71 | p4 | (AAAT)3 | 12 | 120,546 | 120,557 | *rps15-ycf1* | IGS | SSC |
| 72 | p1 | (T)10 | 10 | 120,992 | 121,001 | *ycf1* | CDS | SSC |
| 73 | p1 | (T)15 | 15 | 121,607 | 121,621 | *ycf1* | CDS | SSC |
| 74 | p1 | (T)14 | 14 | 121,820 | 121,833 | *ycf1* | CDS | SSC |
| 75 | p1 | (T)16 | 16 | 122,263 | 122,278 | *ycf1* | CDS | SSC |
| 76 | p1 | (T)11 | 11 | 122,823 | 122,833 | *ycf1* | CDS | SSC |
| 77 | p1 | (A)10 | 10 | 124,023 | 124,032 | *ycf1* | CDS | SSC |
| 78 | p2 | (TC)5 | 10 | 145,591 | 145,600 | *ycf2* | CDS | IRa |
| *Fritillaria taipaiensis* | | | | | | | | |
| 1 | p1 | (T)12 | 12 | 1,678 | 1,689 | *trnK-UUU* | intron | LSC |
| 2 | p1 | (A)16 | 16 | 4,005 | 4,020 | *trnK-UUU-rps16* | IGS | LSC |
| 3 | p1 | (T)14 | 14 | 4,214 | 4,227 | *trnK-UUU-rps16* | IGS | LSC |
| 4 | p1 | (A)18 | 18 | 6,727 | 6,744 | *psbK-psbI* | IGS | LSC |
| 5 | p2 | (AT)7 | 14 | 7,260 | 7,273 | *trnS-GCU-trnG-GCC* | IGS | LSC |
| 6 | p2 | (AT)6 | 12 | 7,277 | 7,288 | *trnS-GCU-trnG-GCC* | IGS | LSC |
| 7 | p1 | (T)12 | 12 | 7,705 | 7,716 | *trnS-GCU-trnG-GCC* | IGS | LSC |
| 8 | p1 | (T)13 | 13 | 8,074 | 8,086 | *trnG-GCC* | intron | LSC |
| 9 | p4 | (AGAA)3 | 12 | 8,721 | 8,732 | *trnG-GCC-trnR-UCU* | IGS | LSC |
| 10 | p2 | (TA)6 | 12 | 8,840 | 8,851 | *trnR-UCU-atpA* | IGS | LSC |
| 11 | p1 | (A)14 | 14 | 11,669 | 11,682 | *atpF* | intron | LSC |
| 12 | p1 | (T)10 | 10 | 12,787 | 12,796 | *atpH-atpI* | IGS | LSC |
| 13 | p1 | (A)16 | 16 | 13,015 | 13,030 | *atpH-atpI* | IGS | LSC |
| 14 | p4 | (AATT)3 | 12 | 14,462 | 14,473 | *atpI-rps2* | IGS | LSC |
| 15 | p1 | (T)17 | 17 | 15,232 | 15,248 | *rps2-rpoC2* | IGS | LSC |
| 16 | p1 | (T)12 | 12 | 17,325 | 17,336 | *rpoC2* | CDS | LSC |
| 17 | p1 | (T)11 | 11 | 17,434 | 17,444 | *rpoC2* | CDS | LSC |
| 18 | p2 | (TA)5 | 10 | 18,800 | 18,809 | *rpoC2* | CDS | LSC |
| 19 | p4 | (TAAT)3 | 12 | 27,826 | 27,837 | *psbM-trnD-GUC* | IGS | LSC |
| 20 | p1 | (A)21 | 21 | 28,160 | 28,180 | *psbM-trnD-GUC* | IGS | LSC |
| 21 | p1 | (T)12 | 12 | 28,485 | 28,496 | *trnD-GUC-trnY-GUA* | IGS | LSC |
| 22 | p1 | (A)11 | 11 | 28,520 | 28,530 | *trnD-GUC-trnY-GUA* | IGS | LSC |
| 23 | p2 | (AT)5 | 10 | 29,042 | 29,051 | *trnE-UUC-trnT-GGU* | IGS | LSC |
| 24 | p1 | (T)17 | 17 | 29,320 | 29,336 | *trnE-UUC-trnT-GGU* | IGS | LSC |
| 25 | p5 | (TATTA)3 | 15 | 29,445 | 29,459 | *trnE-UUC-trnT-GGU* | IGS | LSC |
| 26 | p1 | (A)13 | 13 | 29,651 | 29,663 | *trnT-GGU-psbD* | IGS | LSC |
| 27 | p2 | (AT)5 | 10 | 29,855 | 29,864 | *trnT-GGU-psbD* | IGS | LSC |
| 28 | p2 | (AT)5 | 10 | 30,025 | 30,034 | *trnT-GGU-psbD* | IGS | LSC |
| 29 | p1 | (A)12 | 12 | 33,659 | 33,670 | *psbZ-trnG-UCC* | IGS | LSC |
| 30 | p1 | (T)10 | 10 | 39,345 | 39,354 | *psaA-ycf3* | IGS | LSC |
| 31 | p1 | (A)16 | 16 | 39,839 | 39,854 | *psaA-ycf3* | IGS | LSC |
| 32 | p1 | (A)11 | 11 | 41,598 | 41,608 | *ycf3* | intron | LSC |
| 33 | p2 | (TC)5 | 10 | 41,644 | 41,653 | *ycf3* | intron | LSC |
| 34 | p1 | (A)11 | 11 | 42,635 | 42,645 | *trnS-GGA-rps4* | IGS | LSC |
| 35 | p1 | (A)11 | 11 | 42,681 | 42,691 | *trnS-GGA-rps4* | IGS | LSC |
| 36 | p1 | (T)10 | 10 | 43,894 | 43,903 | *trnT-UGU-trnL-UAA* | IGS | LSC |
| 37 | p1 | (A)13 | 13 | 44,260 | 44,272 | *trnT-UGU-trnL-UAA* | IGS | LSC |
| 38 | p1 | (A)16 | 16 | 44,460 | 44,475 | *trnT-UGU-trnL-UAA* | IGS | LSC |
| 39 | p2 | (AT)6 | 12 | 44,961 | 44,972 | *trnL-UAA* | intron | LSC |
| 40 | p1 | (T)13 | 13 | 45,275 | 45,287 | *trnL-UAA-trnF-GAA* | IGS | LSC |
| 41 | p1 | (A)10 | 10 | 55,943 | 55,952 | *accD-psaI* | IGS | LSC |
| 42 | p1 | (A)16 | 16 | 56,320 | 56,335 | *accD-psaI* | IGS | LSC |
| 43 | p3 | (GAA)4 | 12 | 56,357 | 56,368 | *accD-psaI* | IGS | LSC |
| 44 | p1 | (A)15 | 15 | 58,305 | 58,319 | *cemA* | CDS | LSC |
| 45 | p2 | (AT)5 | 10 | 60,807 | 60,816 | *petA-psbJ* | IGS | LSC |
| 46 | p1 | (A)16 | 16 | 62,522 | 62,537 | *psbE-petL* | IGS | LSC |
| 47 | p1 | (T)13 | 13 | 62,647 | 62,659 | *psbE-petL* | IGS | LSC |
| 48 | p1 | (A)15 | 15 | 63,540 | 63,554 | *trnW-CCA-trnP-UGG* | IGS | LSC |
| 49 | p1 | (A)10 | 10 | 64,243 | 64,252 | *psaJ-rpl33* | IGS | LSC |
| 50 | p1 | (A)15 | 15 | 64,289 | 64,303 | *psaJ-rpl33* | IGS | LSC |
| 51 | p1 | (T)15 | 15 | 67,901 | 67,915 | *clpP* | intron | LSC |
| 52 | p1 | (A)11 | 11 | 68,293 | 68,303 | *clpP* | intron | LSC |
| 53 | p1 | (T)16 | 16 | 68,616 | 68,631 | *clpP* | intron | LSC |
| 54 | p1 | (A)14 | 14 | 72,194 | 72,207 | *petB* | intron | LSC |
| 55 | p2 | (AT)8 | 16 | 75,086 | 75,101 | *petD-rpoA* | IGS | LSC |
| 56 | p4 | (TTCT)3 | 12 | 79,556 | 79,567 | *rpl16* | intron | LSC |
| 57 | p1 | (T)11 | 11 | 79,629 | 79,639 | *rpl16* | intron | LSC |
| 58 | p1 | (T)13 | 13 | 79,942 | 79,954 | *rpl16-rps3* | IGS | LSC |
| 59 | p1 | (A)17 | 17 | 81,184 | 81,200 | *rpl22-rps19* | IGS | LSC |
| 60 | p1 | (T)11 | 11 | 82,634 | 82,644 | *rpl2* | intron | IRb |
| 61 | p2 | (GA)5 | 10 | 86,912 | 86,921 | *ycf2* | CDS | IRb |
| 62 | p1 | (A)10 | 10 | 110,116 | 110,125 | *ndhF-rpl32* | IGS | SSC |
| 63 | p3 | (AAT)5 | 15 | 110,745 | 110,759 | *rpl32-trnL-UAG* | IGS | SSC |
| 64 | p1 | (T)13 | 13 | 111,006 | 111,018 | *rpl33-trnL-UAG* | IGS | SSC |
| 65 | p1 | (A)17 | 17 | 111,063 | 111,079 | *rpl33-trnL-UAG* | IGS | SSC |
| 66 | p4 | (AATA)3 | 12 | 112,846 | 112,857 | *ndhD* | CDS | SSC |
| 67 | p1 | (A)10 | 10 | 115,616 | 115,625 | *ndhG* | CDS | SSC |
| 68 | p4 | (AAAT)4 | 16 | 115,961 | 115,976 | *ndhG-ndhI* | IGS | SSC |
| 69 | p1 | (A)12 | 12 | 116,274 | 116,285 | *ndhG-ndhI* | IGS | SSC |
| 70 | p2 | (TC)5 | 10 | 119,863 | 119,872 | *ndhH* | CDS | SSC |
| 71 | p1 | (T)14 | 14 | 120,878 | 120,891 | *rps15-ycf1* | IGS | SSC |
| 72 | p4 | (AAAT)3 | 12 | 120,933 | 120,944 | *rps15-ycf1* | IGS | SSC |
| 73 | p1 | (T)10 | 10 | 121,379 | 121,388 | *ycf1* | CDS | SSC |
| 74 | p1 | (T)15 | 15 | 121,994 | 122,008 | *ycf1* | CDS | SSC |
| 75 | p1 | (T)14 | 14 | 122,207 | 122,220 | *ycf1* | CDS | SSC |
| 76 | p1 | (T)16 | 16 | 122,650 | 122,665 | *ycf1* | CDS | SSC |
| 77 | p1 | (T)11 | 11 | 123,210 | 123,220 | *ycf1* | CDS | SSC |
| 78 | p2 | (TC)5 | 10 | 146,238 | 146,247 | *ycf2* | CDS | IRa |
| 79 | p1 | (A)11 | 11 | 150,515 | 150,525 | *rpl2* | intron | IRa |
| *Fritillaria yuzhongensis* | | | | | | | | |
| 1 | p1 | (T)14 | 14 | 1,773 | 1,786 | *trnK-UUU* | intron | LSC |
| 2 | p1 | (A)20 | 20 | 4,102 | 4,121 | *trnK-UUU-rps16* | IGS | LSC |
| 3 | p1 | (A)10 | 10 | 4,138 | 4,147 | *trnK-UUU-rps16* | IGS | LSC |
| 4 | p1 | (T)14 | 14 | 4,316 | 4,329 | *trnK-UUU-rps16* | IGS | LSC |
| 5 | p1 | (A)18 | 18 | 6,829 | 6,846 | *psbK-psbI* | IGS | LSC |
| 6 | p2 | (AT)6 | 12 | 7,362 | 7,373 | *trnS-GCU-trnG-GCC* | IGS | LSC |
| 7 | p1 | (T)14 | 14 | 7,785 | 7,798 | *trnS-GCU-trnG-GCC* | IGS | LSC |
| 8 | p1 | (T)12 | 12 | 8,156 | 8,167 | *trnG-GCC* | intron | LSC |
| 9 | p4 | (AGAA)3 | 12 | 8,802 | 8,813 | *trnG-GCC-trnR-UCU* | IGS | LSC |
| 10 | p2 | (TA)7 | 24 | 8,921 | 8,934 | *trnR-UCU-atpA* | IGS | LSC |
| 11 | p1 | (T)10 | 10 | 8,935 | 8,944 | *trnR-UCU-atpA* | IGS | LSC |
| 12 | p1 | (A)14 | 14 | 11,754 | 11,767 | *atpF* | intron | LSC |
| 13 | p1 | (A)12 | 12 | 13,105 | 13,116 | *atpH-atpI* | IGS | LSC |
| 14 | p4 | (AATT)3 | 12 | 14,553 | 14,564 | *atpI-rps2* | IGS | LSC |
| 15 | p1 | (T)14 | 14 | 15,323 | 15,336 | *rps2-rpoC2* | IGS | LSC |
| 16 | p1 | (T)12 | 12 | 17,412 | 17,423 | *rpoC2* | CDS | LSC |
| 17 | p1 | (T)11 | 11 | 17,521 | 17,531 | *rpoC2* | CDS | LSC |
| 18 | p2 | (TA)5 | 10 | 18,887 | 18,896 | *rpoC2* | CDS | LSC |
| 19 | p4 | (TAAT)3 | 12 | 27,913 | 27,924 | *psbM-trnD-GUC* | IGS | LSC |
| 20 | p1 | (A)21 | 21 | 28,247 | 28,267 | *psbM-trnD-GUC* | IGS | LSC |
| 21 | p1 | (T)16 | 16 | 28,572 | 28,587 | *trnD-GUC-trnY-GUA* | IGS | LSC |
| 22 | p1 | (A)10 | 10 | 28,611 | 28,620 | *trnD-GUC-trnY-GUA* | IGS | LSC |
| 23 | p2 | (AT)5 | 10 | 29,157 | 29,166 | *trnE-UUC-trnT-GGU* | IGS | LSC |
| 24 | p1 | (T)10 | 10 | 29,329 | 29,338 | *trnE-UUC-trnT-GGU* | IGS | LSC |
| 25 | p1 | (T)11 | 11 | 29,436 | 29,446 | *trnE-UUC-trnT-GGU* | IGS | LSC |
| 26 | p5 | (TATTA)3 | 15 | 29,555 | 29,569 | *trnE-UUC-trnT-GGU* | IGS | LSC |
| 27 | p1 | (A)12 | 12 | 29,761 | 29,772 | *trnT-GGU-psbD* | IGS | LSC |
| 28 | p2 | (AT)5 | 10 | 29,964 | 29,973 | *trnT-GGU-psbD* | IGS | LSC |
| 29 | p2 | (AT)5 | 10 | 30,134 | 30,143 | *trnT-GGU-psbD* | IGS | LSC |
| 30 | p1 | (A)13 | 13 | 33,756 | 33,768 | *psbZ-trnG-UCC* | IGS | LSC |
| 31 | p1 | (A)16 | 16 | 39,937 | 39,952 | *psaA-ycf3* | IGS | LSC |
| 32 | p1 | (A)11 | 11 | 41,696 | 41,706 | *ycf3* | intron | LSC |
| 33 | p2 | (TC)5 | 10 | 41,742 | 41,751 | *ycf3* | intron | LSC |
| 34 | p1 | (A)10 | 10 | 42,734 | 42,743 | *trnS-GGA-rps4* | IGS | LSC |
| 35 | p1 | (T)11 | 11 | 43,945 | 43,955 | *trnT-UGU-trnL-UAA* | IGS | LSC |
| 36 | p1 | (A)22 | 22 | 44,308 | 44,329 | *trnT-UGU-trnL-UAA* | IGS | LSC |
| 37 | p1 | (A)16 | 16 | 44,517 | 44,532 | *trnT-UGU-trnL-UAA* | IGS | LSC |
| 38 | p2 | (AT)6 | 12 | 45,018 | 45,029 | *trnL-UAA* | intron | LSC |
| 39 | p1 | (T)20 | 20 | 45,332 | 45,351 | *trnL-UAA-trnF-GAA* | IGS | LSC |
| 40 | p1 | (A)14 | 14 | 56,383 | 56,396 | *accD-psaI* | IGS | LSC |
| 41 | p1 | (A)19 | 19 | 58,360 | 58,378 | *cemA* | CDS | LSC |
| 42 | p2 | (AT)5 | 10 | 60,866 | 60,875 | *petA-psbJ* | IGS | LSC |
| 43 | p1 | (A)25 | 25 | 62,581 | 62,605 | *psbE-petL* | IGS | LSC |
| 44 | p1 | (T)13 | 13 | 62,706 | 62,718 | *psbE-petL* | IGS | LSC |
| 45 | p1 | (A)14 | 14 | 63,599 | 63,612 | *trnW-CCA-trnP-UGG* | IGS | LSC |
| 46 | p1 | (A)11 | 11 | 64,304 | 64,314 | *psaJ-rpl33* | IGS | LSC |
| 47 | p1 | (A)16 | 16 | 64,351 | 64,366 | *psaJ-rpl33* | IGS | LSC |
| 48 | p1 | (T)10 | 10 | 67,958 | 67,967 | *clpP* | intron | LSC |
| 49 | p1 | (A)10 | 10 | 68,345 | 68,354 | *clpP* | intron | LSC |
| 50 | p1 | (T)15 | 15 | 68,667 | 68,681 | *clpP* | intron | LSC |
| 51 | p1 | (T)11 | 11 | 71,139 | 71,149 | *psbB-psbT* | IGS | LSC |
| 52 | p1 | (A)11 | 11 | 72,244 | 72,254 | *petB* | CDS | LSC |
| 53 | p2 | (AT)7 | 14 | 75,133 | 75,146 | *petD-rpoA* | IGS | LSC |
| 54 | p4 | (TTCT)3 | 12 | 79,609 | 79,620 | *rpl16* | IGS | LSC |
| 55 | p1 | (T)10 | 10 | 79,682 | 79,691 | *rpl16* | IGS | LSC |
| 56 | p1 | (T)12 | 12 | 79,994 | 80,005 | *rpl16-rps3* | IGS | LSC |
| 57 | p1 | (A)17 | 17 | 81,176 | 81,192 | *rpl22-rps19* | IGS | LSC |
| 58 | p1 | (A)17 | 17 | 81,245 | 81,261 | *rpl22-rps19* | IGS | LSC |
| 59 | p1 | (T)10 | 10 | 82,695 | 82,704 | *rpl2* | intron | IRb |
| 60 | p2 | (GA)5 | 10 | 86,972 | 86,981 | *ycf2* | CDS | IRb |
| 61 | p3 | (AAT)5 | 15 | 110,805 | 110,819 | *rpl32-trnL-UAG* | IGS | SSC |
| 62 | p1 | (T)12 | 12 | 111,066 | 111,077 | *rpl32-trnL-UAG* | IGS | SSC |
| 63 | p1 | (A)17 | 17 | 111,122 | 111,138 | *rpl32-trnL-UAG* | IGS | SSC |
| 64 | p4 | (AATA)3 | 12 | 112,905 | 112,916 | *ndhD* | CDS | SSC |
| 65 | p1 | (A)10 | 10 | 115,675 | 115,684 | *ndhG* | CDS | SSC |
| 66 | p4 | (AAAT)4 | 16 | 116,020 | 116,035 | *ndhG-ndhI* | IGS | SSC |
| 67 | p1 | (A)13 | 13 | 116,333 | 116,345 | *ndhG-ndhI* | IGS | SSC |
| 68 | p2 | (TC)5 | 10 | 119,918 | 119,927 | *ndhH* | CDS | SSC |
| 69 | p1 | (T)15 | 15 | 120,932 | 120,946 | *rps15-ycf1* | IGS | SSC |
| 70 | p4 | (AAAT)3 | 12 | 120,988 | 120,999 | *rps15-ycf1* | IGS | SSC |
| 71 | p1 | (T)10 | 10 | 121,434 | 121,443 | *ycf1* | CDS | SSC |
| 72 | p1 | (T)15 | 15 | 122,040 | 122,054 | *ycf1* | CDS | SSC |
| 73 | p1 | (T)14 | 14 | 122,253 | 122,266 | *ycf1* | CDS | SSC |
| 74 | p1 | (T)10 | 10 | 122,696 | 122,705 | *ycf1* | CDS | SSC |
| 75 | p1 | (T)11 | 11 | 123,250 | 123,260 | *ycf1* | CDS | SSC |
| 76 | p2 | (TC)5 | 10 | 146,272 | 146,281 | *ycf2* | CDS | IRa |
| 77 | p1 | (A)10 | 10 | 150,549 | 150,558 | *rpl2* | intron | IRa |
| *Fritillaria sinica* | | | | | | | | |
| 1 | p1 | (T)12 | 12 | 1,773 | 1,784 | *trnK-UUU* | intron | LSC |
| 2 | p1 | (T)11 | 11 | 2,042 | 2,052 | *matK* | CDS | LSC |
| 3 | p1 | (A)13 | 13 | 4,100 | 4,112 | *trnK-UUU-rps16* | IGS | LSC |
| 4 | p1 | (T)11 | 11 | 4,281 | 4,291 | *trnK-UUU-rps16* | IGS | LSC |
| 5 | p2 | (TA)6 | 12 | 5,603 | 5,614 | *rps16* | intron | LSC |
| 6 | p1 | (T)10 | 10 | 6,135 | 6,144 | *rps16-trnQ-UUG* | IGS | LSC |
| 7 | p1 | (A)18 | 18 | 6,807 | 6,824 | *psbK-psbI* | IGS | LSC |
| 8 | p2 | (AT)6 | 12 | 7,340 | 7,351 | *trnS-GCU-trnG-GCC* | IGS | LSC |
| 9 | p1 | (T)13 | 13 | 7,768 | 7,780 | *trnS-GCU-trnG-GCC* | IGS | LSC |
| 10 | p1 | (T)15 | 15 | 8,134 | 8,148 | *trnG-GCC* | intron | LSC |
| 11 | p2 | (TA)6 | 12 | 8,902 | 8,913 | *trnR-UCU-atpA* | IGS | LSC |
| 12 | p1 | (T)10 | 10 | 8,914 | 8,923 | *trnR-UCU-atpA* | IGS | LSC |
| 13 | p1 | (A)15 | 15 | 11,733 | 11,747 | *atpF* | intron | LSC |
| 14 | p1 | (T)10 | 10 | 12,858 | 12,867 | *atpF-atpI* | IGS | LSC |
| 15 | p5 | (TATTG)3 | 15 | 13,046 | 13,060 | *atpF-atpI* | IGS | LSC |
| 16 | p1 | (A)12 | 12 | 13,091 | 13,102 | *atpF-atpI* | IGS | LSC |
| 17 | p3 | (ATA)4 | 12 | 13,145 | 13,156 | *atpF-atpI* | IGS | LSC |
| 18 | p4 | (AATT)3 | 12 | 14,538 | 14,549 | *atpI-rps2* | IGS | LSC |
| 19 | p1 | (T)11 | 11 | 15,308 | 15,318 | *rps2-rpoC2* | IGS | LSC |
| 20 | p1 | (T)12 | 12 | 17,394 | 17,405 | *rpoC2* | CDS | LSC |
| 21 | p1 | (T)11 | 11 | 17,503 | 17,513 | *rpoC2* | CDS | LSC |
| 22 | p2 | (TA)5 | 10 | 18,869 | 18,878 | *rpoC2* | CDS | LSC |
| 23 | p4 | (TAAT)3 | 12 | 27,939 | 27,950 | *psbM-trnD-GUC* | IGS | LSC |
| 24 | p1 | (A)26 | 26 | 28,269 | 28,294 | *psbM-trnD-GUC* | IGS | LSC |
| 25 | p1 | (T)16 | 16 | 28,599 | 28,614 | *trnD-GUC-trnY-GUA* | IGS | LSC |
| 26 | p1 | (A)16 | 16 | 28,634 | 28,649 | *trnD-GUC-trnY-GUA* | IGS | LSC |
| 27 | p2 | (AT)5 | 10 | 29,163 | 29,172 | *trnE-UUC-trnT-GGU* | IGS | LSC |
| 28 | p1 | (T)16 | 16 | 29,446 | 29,461 | *trnE-UUC-trnT-GGU* | IGS | LSC |
| 29 | p1 | (A)13 | 13 | 29,771 | 29,783 | *trnT-GGU-psbD* | IGS | LSC |
| 30 | p2 | (AT)5 | 10 | 30,137 | 30,146 | *trnT-GGU-psbD* | IGS | LSC |
| 31 | p2 | (AT)5 | 10 | 30,316 | 30,325 | *trnT-GGU-psbD* | IGS | LSC |
| 32 | p1 | (A)10 | 10 | 33,951 | 33,960 | *psbZ-trnG-UCC* | IGS | LSC |
| 33 | p1 | (T)11 | 11 | 39,634 | 39,644 | *psaA-ycf3* | IGS | LSC |
| 34 | p1 | (A)16 | 16 | 40,129 | 40,144 | *psaA-ycf3* | IGS | LSC |
| 35 | p1 | (A)11 | 11 | 41,894 | 41,904 | *ycf3* | intron | LSC |
| 36 | p1 | (A)10 | 10 | 42,929 | 42,938 | *trnS-GGA-rps4* | IGS | LSC |
| 37 | p1 | (A)11 | 11 | 42,974 | 42,984 | *trnS-GGA-rps4* | IGS | LSC |
| 38 | p1 | (T)10 | 10 | 44,193 | 44,202 | *trnT-UGU-trnL-UAA* | IGS | LSC |
| 39 | p1 | (A)23 | 23 | 44,540 | 44,562 | *trnT-UGU-trnL-UAA* | IGS | LSC |
| 40 | p1 | (A)22 | 22 | 44,747 | 44,768 | *trnT-UGU-trnL-UAA* | IGS | LSC |
| 41 | p2 | (AT)6 | 12 | 45,254 | 45,265 | *trnL-UAA* | intron | LSC |
| 42 | p1 | (T)16 | 16 | 45,568 | 45,583 | *trnL-UAA-trnF-GAA* | IGS | LSC |
| 43 | p1 | (T)13 | 13 | 52,148 | 52,160 | *atpB-rbcL* | IGS | LSC |
| 44 | p1 | (A)10 | 10 | 56,247 | 56,256 | *accD-psaI* | IGS | LSC |
| 45 | p1 | (A)16 | 16 | 56,761 | 56,776 | *accD-psaI* | IGS | LSC |
| 46 | p3 | (GAA)4 | 12 | 56,798 | 56,809 | *accD-psaI* | IGS | LSC |
| 47 | p1 | (A)16 | 16 | 58,781 | 58,796 | *accD-psaI* | IGS | LSC |
| 48 | p1 | (T)16 | 16 | 63,117 | 63,132 | *psbE-petL* | IGS | LSC |
| 49 | p1 | (A)15 | 15 | 64,013 | 64,027 | *trnW-CCA-trnP-UGG* | IGS | LSC |
| 50 | p1 | (A)10 | 10 | 64,472 | 64,481 | *trnP-UGG-psaJ* | IGS | LSC |
| 51 | p1 | (A)11 | 11 | 64,717 | 64,727 | *psaJ-rpl33* | IGS | LSC |
| 52 | p1 | (A)10 | 10 | 64,764 | 64,773 | *psaJ-rpl33* | IGS | LSC |
| 53 | p2 | (AT)7 | 14 | 65,013 | 65,026 | *psaJ-rpl34* | IGS | LSC |
| 54 | p1 | (T)11 | 11 | 68,369 | 68,379 | *clpP* | intron | LSC |
| 55 | p1 | (A)11 | 11 | 68,757 | 68,767 | *clpP* | intron | LSC |
| 56 | p1 | (T)12 | 12 | 69,080 | 69,091 | *clpP* | intron | LSC |
| 57 | p1 | (A)16 | 16 | 72,655 | 72,670 | *petB* | intron | LSC |
| 58 | p2 | (AT)7 | 14 | 75,551 | 75,564 | *petD-rpoA* | IGS | LSC |
| 59 | p4 | (TTCT)4 | 16 | 80,019 | 80,034 | *rpl16* | intron | LSC |
| 60 | p1 | (T)19 | 19 | 80,410 | 80,428 | *rpl16-rps3* | IGS | LSC |
| 61 | p1 | (A)22 | 22 | 81,650 | 81,671 | *rpl22-rps19* | IGS | LSC |
| 62 | p2 | (GA)5 | 10 | 87,382 | 87,391 | *ycf2* | CDS | IRb |
| 63 | p1 | (T)12 | 12 | 111,468 | 111,479 | *rpl32-trnL-UAG* | IGS | SSC |
| 64 | p1 | (A)13 | 13 | 111,524 | 111,536 | *rpl32-trnL-UAG* | IGS | SSC |
| 65 | p4 | (AATA)3 | 12 | 113,305 | 113,316 | *ndhD* | CDS | SSC |
| 66 | p1 | (A)10 | 10 | 116,075 | 116,084 | *ndhG* | CDS | SSC |
| 67 | p4 | (AAAT)4 | 16 | 116,420 | 116,435 | *ndhG-ndhI* | IGS | SSC |
| 68 | p2 | (TC)5 | 10 | 120,315 | 120,324 | *ndhH* | CDS | SSC |
| 69 | p1 | (T)17 | 17 | 121,330 | 121,346 | *rps15-ycf1* | IGS | SSC |
| 70 | p4 | (AAAT)3 | 12 | 121,387 | 121,398 | *rps15-ycf1* | IGS | SSC |
| 71 | p1 | (T)10 | 10 | 121,833 | 121,842 | *ycf1* | CDS | SSC |
| 72 | p1 | (T)15 | 15 | 122,448 | 122,462 | *ycf1* | CDS | SSC |
| 73 | p1 | (T)14 | 14 | 122,661 | 122,674 | *ycf1* | CDS | SSC |
| 74 | p1 | (T)16 | 16 | 123,104 | 123,119 | *ycf1* | CDS | SSC |
| 75 | p1 | (T)11 | 11 | 123,664 | 123,674 | *ycf1* | CDS | SSC |
| 76 | p2 | (TC)5 | 10 | 146,691 | 146,700 | *ycf2* | CDS | IRa |
| *Fritillaria dajinensis* | | | | | | | | |
| 1 | p1 | (T)18 | 18 | 1,679 | 1,696 | *trnK-UUU* | intron | LSC |
| 2 | p1 | (T)10 | 10 | 3,599 | 3,608 | *trnK-UUU* | intron | LSC |
| 3 | p1 | (A)14 | 14 | 4,013 | 4,026 | *trnK-UUU-rps16* | IGS | LSC |
| 4 | p1 | (T)11 | 11 | 4,220 | 4,230 | *trnK-UUU-rps16* | IGS | LSC |
| 5 | p1 | (A)19 | 19 | 6,751 | 6,769 | *psbK-psbI* | IGS | LSC |
| 6 | p2 | (AT)6 | 12 | 7,285 | 7,296 | *trnS-GCU-trnG-GCC* | IGS | LSC |
| 7 | p1 | (T)15 | 15 | 7,714 | 7,728 | *trnS-GCU-trnG-GCC* | IGS | LSC |
| 8 | p1 | (T)11 | 11 | 8,086 | 8,096 | *trnG-GCC* | IGS | LSC |
| 9 | p4 | (AGAA)3 | 12 | 8,732 | 8,743 | *trnG-GCC-trnR-UCU* | IGS | LSC |
| 10 | p2 | (TA)6 | 12 | 8,851 | 8,862 | *trnR-UCU-atpA* | IGS | LSC |
| 11 | p1 | (A)15 | 15 | 11,678 | 11,692 | *atpF* | intron | LSC |
| 12 | p1 | (A)11 | 11 | 13,030 | 13,040 | *atpH-atpI* | IGS | LSC |
| 13 | p4 | (AATT)3 | 12 | 14,479 | 14,490 | *atpI-rps2* | IGS | LSC |
| 14 | p1 | (T)16 | 16 | 15,249 | 15,264 | *atpI-rps2* | IGS | LSC |
| 15 | p1 | (T)12 | 12 | 17,340 | 17,351 | *rpoC2* | CDS | LSC |
| 16 | p1 | (T)11 | 11 | 17,449 | 17,459 | *rpoC2* | CDS | LSC |
| 17 | p2 | (TA)5 | 10 | 18,815 | 18,824 | *rpoC2* | CDS | LSC |
| 18 | p4 | (TAAT)3 | 12 | 27,860 | 27,871 | *psbM-trnD-GUC* | IGS | LSC |
| 19 | p1 | (A)19 | 19 | 28,194 | 28,212 | *psbM-trnD-GUC* | IGS | LSC |
| 20 | p1 | (T)14 | 14 | 28,517 | 28,530 | *trnD-GUC-trnY-GUA* | IGS | LSC |
| 21 | p2 | (AT)6 | 12 | 28,558 | 28,569 | *trnD-GUC-trnY-GUA* | IGS | LSC |
| 22 | p2 | (AT)5 | 10 | 29,070 | 29,079 | *trnE-UUC-trnT-GGU* | IGS | LSC |
| 23 | p1 | (T)11 | 11 | 29,242 | 29,252 | *trnE-UUC-trnT-GGU* | IGS | LSC |
| 24 | p1 | (T)16 | 16 | 29,350 | 29,365 | *trnE-UUC-trnT-GGU* | IGS | LSC |
| 25 | p5 | (TATTA)3 | 15 | 29,474 | 29,488 | *trnE-UUC-trnT-GGU* | IGS | LSC |
| 26 | p1 | (A)12 | 12 | 29,680 | 29,691 | *trnT-GGU-psbD* | IGS | LSC |
| 27 | p2 | (AT)5 | 10 | 30,047 | 30,056 | *trnT-GGU-psbD* | IGS | LSC |
| 28 | p2 | (AT)5 | 10 | 30,235 | 30,244 | *trnT-GGU-psbD* | IGS | LSC |
| 29 | p1 | (A)11 | 11 | 33,869 | 33,879 | *psbZ-trnG-UCC* | IGS | LSC |
| 30 | p1 | (T)11 | 11 | 39,555 | 39,565 | *psaA-ycf3* | IGS | LSC |
| 31 | p1 | (A)16 | 16 | 40,050 | 40,065 | *psaA-ycf3* | IGS | LSC |
| 32 | p1 | (A)10 | 10 | 41,814 | 41,823 | *ycf3* | intron | LSC |
| 33 | p2 | (TC)5 | 10 | 41,861 | 41,870 | *ycf3* | intron | LSC |
| 34 | p1 | (T)14 | 14 | 44,063 | 44,076 | *trnT-UGU-trnL-UAA* | IGS | LSC |
| 35 | p1 | (A)16 | 16 | 44,434 | 44,449 | *trnT-UGU-trnL-UAA* | IGS | LSC |
| 36 | p1 | (A)25 | 25 | 44,633 | 44,657 | *trnT-UGU-trnL-UAA* | IGS | LSC |
| 37 | p2 | (AT)6 | 12 | 45,143 | 45,154 | *trnL-UAA* | intron | LSC |
| 38 | p1 | (T)11 | 11 | 45,457 | 45,467 | *trnL-UAA-trnF-GAA* | IGS | LSC |
| 39 | p1 | (A)16 | 16 | 56,126 | 56,141 | *accD-psaI* | IGS | LSC |
| 40 | p1 | (A)13 | 13 | 56,655 | 56,667 | *accD-psaI* | IGS | LSC |
| 41 | P3 | (GAA)4 | 12 | 56,689 | 56,700 | *accD-psaI* | IGS | LSC |
| 42 | p1 | (A)15 | 15 | 58,627 | 58,641 | *cemA* | CDS | LSC |
| 43 | p2 | (AT)5 | 10 | 61,134 | 61,143 | *petA-psbJ* | IGS | LSC |
| 44 | p1 | (A)13 | 13 | 62,839 | 62,851 | *psbE-petL* | IGS | LSC |
| 45 | p1 | (T)14 | 14 | 62,958 | 62,971 | *psbE-petL* | IGS | LSC |
| 46 | p1 | (A)16 | 16 | 63,852 | 63,867 | *trnW-CCA-trnP-UGG* | IGS | LSC |
| 47 | p1 | (A)15 | 15 | 64,561 | 64,575 | *psaJ-rpl33* | IGS | LSC |
| 48 | p1 | (A)11 | 11 | 64,612 | 64,622 | *psaJ-rpl33* | IGS | LSC |
| 49 | p1 | (T)12 | 12 | 68,213 | 68,224 | *clpP* | intron | LSC |
| 50 | p1 | (A)13 | 13 | 68,602 | 68,614 | *clpP* | intron | LSC |
| 51 | p1 | (T)13 | 13 | 68,927 | 68,939 | *clpP* | intron | LSC |
| 52 | p1 | (A)11 | 11 | 72,502 | 72,512 | *psbB* | CDS | LSC |
| 53 | p2 | (AT)7 | 14 | 75,375 | 75,388 | *petD-rpoA* | IGS | LSC |
| 54 | p3 | (TTCT)3 | 12 | 79,844 | 79,855 | *rpl16* | intron | LSC |
| 55 | p1 | (T)11 | 11 | 79,917 | 79,927 | *rpl16* | intron | LSC |
| 56 | p1 | (T)14 | 14 | 80,222 | 80,235 | *rpl16* | intron | LSC |
| 57 | p2 | (GA)5 | 10 | 871,82 | 87,191 | *ycf2* | CDS | IRb |
| 58 | p1 | (A)10 | 10 | 110,400 | 110,409 | *ndhF-rpl32* | IGS | SSC |
| 59 | p1 | (T)18 | 18 | 111,284 | 111,301 | *rpl32-trnL-UAG* | IGS | SSC |
| 60 | p1 | (A)20 | 20 | 111,346 | 111,365 | *rpl32-trnL-UAG* | IGS | SSC |
| 61 | p4 | (AATA)3 | 12 | 113,131 | 113,142 | *ndhD* | CDS | SSC |
| 62 | p1 | (A)10 | 10 | 115,901 | 115,910 | *ndhG* | CDS | SSC |
| 63 | p4 | (AAAT)4 | 16 | 116,246 | 116,261 | *ndhG-ndhI* | IGS | SSC |
| 64 | p1 | (A)11 | 11 | 116,559 | 116,569 | *ndhG-ndhI* | IGS | SSC |
| 65 | p2 | (TC)5 | 10 | 120,141 | 120,150 | *ndhH* | CDS | SSC |
| 66 | p1 | (T)14 | 14 | 121,156 | 121,169 | *rps15-ycf1* | IGS | SSC |
| 67 | p4 | (AAAT)3 | 12 | 121,211 | 121,222 | *rps15-ycf1* | IGS | SSC |
| 68 | p1 | (T)10 | 10 | 121,657 | 121,666 | *ycf1* | CDS | SSC |
| 69 | p1 | (T)15 | 15 | 122,272 | 122,286 | *ycf1* | CDS | SSC |
| 70 | p1 | (T)14 | 14 | 122,485 | 122,498 | *ycf1* | CDS | SSC |
| 71 | p1 | (T)10 | 10 | 122,543 | 122,552 | *ycf1* | CDS | SSC |
| 72 | p1 | (T)10 | 10 | 122,928 | 122,937 | *ycf1* | CDS | SSC |
| 73 | p1 | (T)11 | 11 | 123,482 | 123,492 | *ycf1* | CDS | SSC |
| 74 | p1 | (T)12 | 12 | 125,206 | 125,217 | *ycf1* | CDS | SSC |
| 75 | p2 | (TC)5 | 10 | 146,524 | 146,533 | *ycf2* | CDS | IRa |
