# Supplementary material for: Phylogenetic analysis of Fritillaria cirrhosa D. Don and its closely related species based on complete chloroplast genomes
Source: PeerJ. 2019 Aug 21;7:e7480. doi: 10.7717/peerj.7480 (PMC6708372; doi:10.7717/peerj.7480)
Supplement: Table S4 [file peerj-07-7480-s006.docx]

Table S4. Regions of tandem repeat in eight *Fritillaria* chloroplast genomes

|  | LSC | SSC | IR | *trnK-UUU* | *rps11* | *rpl16* | *ycf2* | *ycf1* |
| --- | --- | --- | --- | --- | --- | --- | --- | --- |
| *F. cirrhosa* | 16 | 3 | 5 | 1 | 1 | 1 | 5 | 2 |
| *F. sichuanica* | 19 | 3 | 2 | 1 | 1 | 1 | 2 | 2 |
| *F. przewalskii* | 17 | 3 | 5 | 1 | 1 | 1 | 5 | 2 |
| *F. unibracteata* | 17 | 3 | 5 | 1 | 1 | 1 | 5 | 2 |
| *F. taipaiensis* | 17 | 3 | 5 | 1 | 1 | 1 | 5 | 2 |
| *F. yuzhongensis* | 16 | 3 | 5 | 1 | 1 | 1 | 5 | 2 |
| *F. sinica* | 18 | 3 | 5 | 1 | 1 | 1 | 5 | 2 |
| *F. dajinensis* | 19 | 3 | 5 | 1 | 1 | 1 | 5 | 2 |
