# Supplementary material for: Phylogenetic analysis of Fritillaria cirrhosa D. Don and its closely related species based on complete chloroplast genomes
Source: PeerJ. 2019 Aug 21;7:e7480. doi: 10.7717/peerj.7480 (PMC6708372; doi:10.7717/peerj.7480)
Supplement: Table S5 [file peerj-07-7480-s007.docx]

Table S5. A list of repeated sequences and their locations identified in the eight *Fritillaria* chloroplast genomes.

| NO. | Repeat type | Position A | Length(bp) | Locus | Region | Position B | Length(bp) | Locus | Region |
| --- | --- | --- | --- | --- | --- | --- | --- | --- | --- |
| *Fritillaria cirrhosa* | | | | | | | | | |
| 1 | P | 5,329 | 34 | *rps16* | intron | 5,329 | 34 | *rps16* | intron |
| 2 | F | 7,000 | 31 | *psbI-trnS-GCU* | IGS | 33,240 | 31 | *psbC-trnS-UGA* | IGS |
| 3 | P | 7,002 | 31 | *psbI-trnS-GCU* | LSC | 42,590 | 31 | *trnS-UGA* | LSC |
| 4 | F | 8,526 | 30 | *trnG-GCC* | LSC | 34,040 | 30 | *trnG-UCC* | LSC |
| 5 | P | 26,804 | 38 | *trnC-GCA-petN* | IGS | 26,804 | 38 | *trnC-GCA-petN* | IGS |
| 6 | P | 27,028 | 65 | *petN-psbM* | IGS | 27,028 | 65 | *petN-psbM* | IGS |
| 7 | F | 30,151 | 38 | *trnT-GGU-psbD* | IGS | 30,164 | 38 | *trnT-GGU-psbD* | IGS |
| 8 | P | 33,239 | 32 | *psbC-trnS-UGA* | IGS | 42,592 | 32 | *trnS-GGA* | LSC |
| 9 | F | 36,259 | 50 | *psaB* | LSC | 38,483 | 50 | *psaA* | LSC |
| 10 | F | 36,277 | 32 | *psaB* | LSC | 38,501 | 32 | *psaA* | LSC |
| 11 | F | 41,146 | 39 | *ycf3* | intron | 96,338 | 39 | *rps12-trnV-GAC* | IGS |
| 12 | P | 41,146 | 39 | *ycf3* | LSC | 137,376 | 39 | *trnV-GAC-rps12* | IGS |
| 13 | C | 44,006 | 31 | *trnT-UGU-trnL-UAA* | IGS | 64,811 | 31 | *psaJ-rpl33* | IGS |
| 14 | R | 44,033 | 30 | *trnT-UGU-trnL-UAA* | IGS | 44,033 | 30 | *trnT-UGU-trnL-UAA* | IGS |
| 15 | P | 44,130 | 33 | *trnT-UGU-trnL-UAA* | IGS | 44,130 | 33 | *trnT-UGU-trnL-UAA* | IGS |
| 16 | F | 58,418 | 30 | *ycf4-cemA* | IGS | 58,447 | 30 | *ycf4-cemA* | IGS |
| 17 | R | 78,101 | 31 | *rpl16* | LSC | 78,101 | 31 | *rpl16* | LSC |
| 18 | F | 86,592 | 30 | *ycf2* | IRb | 86,613 | 30 | *ycf2* | IRb |
| 19 | P | 86,592 | 30 | *ycf2* | IRb | 147,110 | 30 | *ycf2* | IRa |
| 20 | P | 86,613 | 30 | *ycf2* | IRb | 147,131 | 30 | *ycf2* | IRa |
| 21 | F | 88,932 | 57 | *ycf2* | IRb | 88,956 | 57 | *ycf2* | IRb |
| 22 | P | 88,932 | 57 | *ycf2* | IRb | 144,740 | 57 | *ycf2* | IRa |
| 23 | F | 88,932 | 37 | *ycf2* | IRb | 88,980 | 37 | *ycf2* | IRb |
| 24 | P | 88,932 | 37 | *ycf2* | IRb | 144,736 | 37 | *ycf2* | IRa |
| 25 | P | 88,956 | 57 | *ycf2* | IRb | 144,764 | 57 | *ycf2* | IRa |
| 26 | F | 88,961 | 32 | *ycf2* | IRb | 88,985 | 32 | *ycf2* | IRb |
| 27 | P | 88,961 | 32 | *ycf2* | IRb | 144,736 | 32 | *ycf2* | IRa |
| 28 | P | 88,980 | 37 | *ycf2* | IRb | 144,784 | 37 | *ycf2* | IRa |
| 29 | P | 88,985 | 32 | *ycf2* | IRb | 144,760 | 32 | *ycf2* | IRa |
| 30 | P | 91,814 | 35 | *ycf15-trnL-CAA* | IGS | 91,814 | 35 | *ycf15-trnL-CAA* | IGS |
| 31 | F | 91,814 | 35 | *ycf15-trnL-CAA* | IGS | 141,904 | 35 | *trnL-CAA-ycf15* | IGS |
| 32 | P | 112,379 | 34 | *ccsA* | SSC | 112,379 | 34 | *ccsA* | SSC |
| 33 | P | 112,800 | 30 | *ccsA-ndhD* | IGS | 112,843 | 30 | *ccsA-ndhD* | IGS |
| 34 | R | 112,925 | 31 | *ccsA-ndhD* | IGS | 112,925 | 31 | *ccsA-ndhD* | IGS |
| 35 | P | 141,904 | 35 | *trnL-CAA-ycf15* | IGS | 141,904 | 35 | *trnL-CAA-ycf15* | IGS |
| 36 | F | 144,736 | 37 | *ycf2* | IRa | 144,784 | 37 | *ycf2* | IRa |
| 37 | F | 144,740 | 57 | *ycf2* | IRa | 144,764 | 57 | *ycf2* | IRa |
| 38 | F | 144,760 | 37 | *ycf2* | IRa | 144,784 | 37 | *ycf2* | IRa |
| 39 | F | 144,769 | 31 | *ycf2* | IRa | 144,793 | 31 | *ycf2* | IRa |
| 40 | F | 147,113 | 30 | *ycf2* | IRa | 147,134 | 30 | *ycf2* | IRa |
| *Fritillaria sichuanica* | | | | | | | | | |
| 1 | P | 1 | 95 | *trnH-psbA* | IGS | 81,821 | 95 | *rps19* | LSC |
| 2 | P | 5,470 | 34 | *rps16* | intron | 5,470 | 34 | *rps16* | intron |
| 3 | F | 7,145 | 31 | *psbI-trnS-GCU* | IGS | 33,450 | 31 | *psbC-trnS-UGA* | IGS |
| 4 | P | 7,147 | 31 | *psbI-trnS-GCU* | IGS | 42,802 | 31 | *trnS-GGA* | LSC |
| 5 | F | 8,681 | 30 | *trnG-GCC* | LSC | 34,252 | 30 | *trnG-UCC* | LSC |
| 6 | F | 8,828 | 47 | *trnG-GCC-trnR-UCU* | IGS | 8,875 | 47 | *trnG-GCC-trnR-UCU* | IGS |
| 7 | P | 26,987 | 38 | *trnC-GCA-petN* | IGS | 26,987 | 38 | *trnC-GCA -petN* | IGS |
| 8 | P | 27,211 | 65 | *petN-petM* | IGS | 27,211 | 65 | *petN-psbM* | IGS |
| 9 | F | 28,341 | 30 | *petM-trnD-GUC* | IGS | 111,425 | 30 | *rpl32-trnL-UAG* | IGS |
| 10 | F | 30,340 | 36 | *trnT-GGU-psbD* | IGS | 30,371 | 36 | *trnT-GGU-psbD* | IGS |
| 11 | F | 30,358 | 30 | *trnT-GGU-psbD* | IGS | 30,367 | 30 | *trnT-GGU-psbD* | IGS |
| 12 | P | 33,449 | 32 | *trnS-UGA* | LSC | 42,804 | 32 | *trnS-GGA* | LSC |
| 13 | F | 36,472 | 50 | *psaB* | LSC | 38,696 | 50 | *psaA* | LSC |
| 14 | F | 36,495 | 30 | *psaB* | LSC | 38,719 | 30 | *psaA* | LSC |
| 15 | F | 41,355 | 39 | *ycf3* | intron | 96,394 | 39 | *rps12-trnV-GAC* | IGS |
| 16 | P | 41,355 | 39 | *ycf3* | intron | 137,441 | 39 | *trnV-GAC-rps12* | IGS |
| 17 | C | 44,174 | 31 | *trnT-UGU-trnL-UAA* | IGS | 64,921 | 31 | *psaJ-rpl33* | IGS |
| 18 | R | 44,201 | 30 | *trnT-UGU-trnL-UAA* | IGS | 44,201 | 30 | *trnT-UGU-trnL-UAA* | IGS |
| 19 | P | 44,298 | 33 | *trnT-UGU-trnL-UAA* | IGS | 44,298 | 33 | *trnT-UGU-trnL-UAA* | IGS |
| 20 | R | 64,698 | 30 | *psaJ-rpl33* | IGS | 111,416 | 30 | *rpl32-trnL-UAG* | IGS |
| 21 | R | 78,186 | 31 | *rps8-rpl14* | IGS | 78,186 | 31 | *rps8-rpl14* | IGS |
| 22 | F | 80,312 | 30 | *rpl16-rps3* | IGS | 111,352 | 30 | *rpl32-trnL-UAG* | IGS |
| 23 | F | 88,991 | 57 | *ycf2* | IRb | 89,015 | 57 | *ycf2* | IRb |
| 24 | P | 88,991 | 57 | *ycf2* | IRb | 144,802 | 57 | *ycf2* | IRa |
| 25 | F | 88,991 | 37 | *ycf2* | IRb | 89,039 | 37 | *ycf2* | IRb |
| 26 | P | 88,991 | 37 | *ycf2* | IRb | 144,798 | 37 | *ycf2* | IRa |
| 27 | P | 89,015 | 57 | *ycf2* | IRb | 144,826 | 57 | *ycf2* | IRa |
| 28 | F | 89,020 | 32 | *ycf2* | IRb | 89,044 | 32 | *ycf2* | IRb |
| 29 | P | 89,020 | 32 | *ycf2* | IRb | 144,798 | 32 | *ycf2* | IRa |
| 30 | P | 89,039 | 37 | *ycf2* | IRb | 144,846 | 37 | *ycf2* | IRa |
| 31 | P | 89,044 | 32 | *ycf2* | IRb | 144,822 | 32 | *ycf2* | IRa |
| 32 | P | 91,873 | 35 | *ycf15-trnL-CAA* | IGS | 91,873 | 35 | *ycf15-trnL-CAA* | IGS |
| 33 | F | 91,873 | 35 | *ycf15-trnL-CAA* | IGS | 141,966 | 35 | *trnL-CAA-ycf15* | IGS |
| 34 | P | 112,451 | 34 | *ccsA* | SSC | 112,451 | 34 | *ccsA* | SSC |
| 35 | P | 112,872 | 30 | *ccsA-ndhD* | IGS | 112,915 | 30 | *ccsA-ndhD* | IGS |
| 36 | R | 112,997 | 31 | *ccsA-ndhD* | IGS | 112,997 | 31 | *ccsA-ndhD* | IGS |
| 37 | P | 141,966 | 35 | *trnL-CAA-ycf15* | IGS | 141,966 | 35 | *trnL-CAA-ycf15* | IGS |
| 38 | F | 144,798 | 37 | *ycf2* | IRa | 144,846 | 37 | *ycf2* | IRa |
| 39 | F | 144,802 | 57 | *ycf2* | IRa | 144,826 | 57 | *ycf2* | IRa |
| 40 | F | 144,822 | 37 | *ycf2* | IRa | 144,846 | 37 | *ycf2* | IRa |
| 41 | F | 144,831 | 31 | *ycf2* | IRa | 144,855 | 31 | *ycf2* | IRa |
| *Fritillaria przewalskii* | | | | | | | | | |
| 1 | P | 1 | 95 | *trnH-psbA* | IGS | 81,839 | 95 | *rps19* | LSC |
| 2 | P | 5,442 | 34 | *rps16* | intron | 5,442 | 34 | *rps16* | intron |
| 3 | F | 7,114 | 31 | *psbI-trnS-GCU* | IGS | 33,377 | 31 | *trnS-UGA* | LSC |
| 4 | P | 7,116 | 31 | *psbI-trnS-GCU* | IGS | 42,735 | 31 | *trnS-GGA* | LSC |
| 5 | F | 8,653 | 30 | *trnG-GCC* | LSC | 34,184 | 30 | *trnG-UCC* | LSC |
| 6 | P | 26,933 | 38 | *trnC-GCA-petN* | IGS | 26,933 | 38 | *trnC-GCA-petN* | IGS |
| 7 | P | 27,157 | 65 | *petN-psbM* | IGS | 27,157 | 65 | *petN-psbM* | IGS |
| 8 | R | 28,275 | 30 | *psbM-trnD-GUC* | IGS | 64,694 | 30 | *psaJ-rpl33* | IGS |
| 9 | F | 30,288 | 38 | *trnT-GGU-psbD* | IGS | 30,301 | 38 | *trnT-GGU-psbD* | IGS |
| 10 | P | 33,376 | 32 | *trnS-UGA* | LSC | 42,737 | 32 | *trnS-GGA* | LSC |
| 11 | F | 36,403 | 50 | *psaB* | LSC | 38,627 | 50 | *psaA* | LSC |
| 12 | F | 36,421 | 32 | *psaB* | LSC | 38,645 | 32 | *psaA* | LSC |
| 13 | F | 41,291 | 39 | *ycf3* | intron | 96,418 | 39 | *rps12-trnV-GAC* | IGS |
| 14 | P | 41,291 | 39 | *ycf3* | intron | 137,460 | 39 | *trnV-GAC-rps12* | IGS |
| 15 | C | 44,153 | 31 | *trnT-UGU-trnL-UAA* | IGS | 64,920 | 31 | *psaJ-rpl33* | IGS |
| 16 | P | 44,276 | 33 | *trnT-UGU-trnL-UAA* | IGS | 44,276 | 33 | *trnT-UGU-trnL-UAA* | IGS |
| 17 | F | 58,568 | 30 | *ycf4-cemA* | IGS | 58,597 | 30 | *ycf4-cemA* | IGS |
| 18 | R | 78,207 | 31 | *rps8-rpl14* | IGS | 78,207 | 31 | *rps8-rpl14* | IGS |
| 19 | F | 86,676 | 30 | *ycf2* | IRb | 86,697 | 30 | *ycf2* | IRb |
| 20 | P | 86,676 | 30 | *ycf2* | IRb | 147,190 | 30 | *ycf2* | IRa |
| 21 | P | 86,697 | 30 | *ycf2* | IRb | 147,211 | 30 | *ycf2* | IRa |
| 22 | F | 89,016 | 57 | *ycf2* | IRb | 89,040 | 57 | *ycf2* | IRb |
| 23 | P | 89,016 | 57 | *ycf2* | IRb | 144,820 | 57 | *ycf2* | IRa |
| 24 | F | 89,016 | 37 | *ycf2* | IRb | 89,064 | 37 | *ycf2* | IRb |
| 25 | P | 89,016 | 37 | *ycf2* | IRb | 144,816 | 37 | *ycf2* | IRa |
| 26 | P | 89,040 | 57 | *ycf2* | IRb | 144,844 | 57 | *ycf2* | IRa |
| 27 | F | 89,045 | 32 | *ycf2* | IRb | 89,069 | 32 | *ycf2* | IRb |
| 28 | P | 89,045 | 32 | *ycf2* | IRb | 144,816 | 32 | *ycf2* | IRa |
| 29 | P | 89,064 | 37 | *ycf2* | IRb | 144,864 | 37 | *ycf2* | IRa |
| 30 | P | 89,069 | 32 | *ycf2* | IRb | 144,840 | 32 | *ycf2* | IRa |
| 31 | P | 91,898 | 35 | *ycf15-trnL-CAA* | IRb | 91,898 | 35 | *ycf15-trnL-CAA* | IRb |
| 32 | F | 91,898 | 35 | *ycf16-trnL-CAA* | IRb | 141,984 | 35 | *ndhB-trnL-CAA* | IGS |
| 33 | P | 112,465 | 34 | *ccsA* | SSC | 112,465 | 34 | *ccsA* | SSC |
| 34 | P | 112,886 | 30 | *ccsA-ndhD* | IGS | 112,929 | 30 | *ccsA-ndhD* | IGS |
| 35 | R | 113,011 | 31 | *ndhD* | SSC | 113,011 | 31 | *ndhD* | SSC |
| 36 | P | 141,984 | 35 | *ycf2* | IRa | 141,984 | 35 | *ycf2* | IRa |
| 37 | F | 144,816 | 37 | *ycf2* | IRa | 144,864 | 37 | *ycf2* | IRa |
| 38 | F | 144,820 | 57 | *ycf2* | IRa | 144,844 | 57 | *ycf2* | IRa |
| 39 | F | 144,840 | 37 | *ycf2* | IRa | 144,864 | 37 | *ycf2* | IRa |
| 40 | F | 144,849 | 31 | *ycf2* | IRa | 144,873 | 31 | *ycf2* | IRa |
| 41 | F | 147,193 | 30 | *ycf2* | IRa | 147,214 | 30 | *ycf2* | IRa |
| *Fritillaria unibracteata* | | | | | | | | | |
| 1 | P | 5,385 | 34 | *rps16* | intron | 5,385 | 34 | *rps16* | intron |
| 2 | F | 7,053 | 31 | *psbI-trnS-GCU* | IGS | 33,318 | 31 | *psbC-trnS-UGA* | IGS |
| 3 | P | 7,055 | 31 | *psbI-trnS-GCU* | IGS | 42,679 | 31 | *trnS-GGA* | LSC |
| 4 | F | 7,658 | 30 | *trnS-GCU-trnG-GCC* | IGS | 7,672 | 30 | *trnS-GCU-trnG-GCC* | IGS |
| 5 | C | 7,720 | 30 | *trnS-GCU-trnG-GCC* | IGS | 28,204 | 30 | *psbM-trnD-GUC* | IGS |
| 6 | C | 7,724 | 31 | *trnS-GCU-trnG-GCC* | IGS | 58,214 | 31 | *cemA* | LSC |
| 7 | F | 8,609 | 30 | *trnG-GCC* | LSC | 34,120 | 30 | *trnG-UCC* | LSC |
| 8 | P | 26,866 | 38 | *trnC-GCA-petN* | IGS | 26,866 | 38 | *trnC-GCA-petN* | IGS |
| 9 | P | 27,090 | 65 | *petN-psbM* | IGS | 27,090 | 65 | *petN-psbM* | IGS |
| 10 | F | 30,217 | 36 | *trnT-GGU-psbD* | IGS | 30,239 | 36 | *trnT-GGU-psbD* | IGS |
| 11 | P | 33,317 | 32 | *psbC-trnS-UGA* | IGS | 42,681 | 32 | *trnS-GGA* | LSC |
| 12 | F | 36,340 | 50 | *psaB* | LSC | 38,564 | 50 | *psaA* | LSC |
| 13 | F | 36,363 | 30 | *psaB* | LSC | 38,587 | 30 | *psaA* | LSC |
| 14 | F | 41,228 | 39 | *ycf3* | intron | 95,658 | 39 | *rps12-trnV-GAC* | IGS |
| 15 | P | 41,228 | 39 | *ycf3* | intron | 136,700 | 39 | *trnV-GAC-rps12* | IGS |
| 16 | C | 44,052 | 31 | *trnT-UGU-trnL-UAA* | IGS | 64,424 | 31 | *psaJ-rpl33* | IGS |
| 17 | R | 44,079 | 33 | *trnT-UGU-trnL-UAA* | IGS | 44,079 | 33 | *trnT-UGU-trnL-UAA* | IGS |
| 18 | P | 44,180 | 33 | *trnT-UGU-trnL-UAA* | IGS | 44,180 | 33 | *trnT-UGU-trnL-UAA* | IGS |
| 19 | F | 44,461 | 30 | *trnT-UGU-trnL-UAA* | IGS | 58,219 | 30 | *cemA* | LSC |
| 20 | F | 44,652 | 30 | *trnT-UGU-trnL-UAA* | IGS | 58,207 | 30 | *cemA* | LSC |
| 21 | R | 64,201 | 30 | *psaJ-rpl33* | IGS | 110,671 | 30 | *rpl32-trnL-UAG* | IGS |
| 22 | R | 77,697 | 31 | *rps8-rpl14* | IGS | 77,697 | 31 | *rps8-rpl14* | IGS |
| 23 | F | 86,175 | 30 | *ycf2* | IRb | 86,196 | 30 | *ycf2* | IRb |
| 24 | P | 86,175 | 30 | *ycf2* | IRb | 146,171 | 30 | *ycf2* | IRa |
| 25 | P | 86,196 | 30 | *ycf2* | IRb | 146,192 | 30 | *ycf2* | IRa |
| 26 | F | 88,515 | 57 | *ycf2* | IRb | 88,539 | 57 | *ycf2* | IRb |
| 27 | P | 88,515 | 57 | *ycf2* | IRb | 143,801 | 57 | *ycf2* | IRa |
| 28 | F | 88,515 | 37 | *ycf2* | IRb | 88,563 | 37 | *ycf2* | IRb |
| 29 | P | 88,515 | 37 | *ycf2* | IRb | 143,797 | 37 | *ycf2* | IRa |
| 30 | P | 88,539 | 57 | *ycf2* | IRb | 143,825 | 57 | *ycf2* | IRa |
| 31 | F | 88,544 | 32 | *ycf2* | IRb | 88,568 | 32 | *ycf2* | IRb |
| 32 | P | 88,544 | 32 | *ycf2* | IRb | 143,797 | 32 | *ycf2* | IRa |
| 33 | P | 88,563 | 37 | *ycf2* | IRb | 143,845 | 37 | *ycf2* | IRa |
| 34 | P | 88,568 | 32 | *ycf2* | IRb | 143,821 | 32 | *ycf2* | IRa |
| 35 | P | 91,137 | 35 | *ycf15-trnL-CAA* | IRb | 91,137 | 35 | *ycf15-trnL-CAA* | IRb |
| 36 | F | 91,137 | 35 | *ycf16-trnL-CAA* | IRb | 141,225 | 35 | *trnL-CAA-ycf15* | IGS |
| 37 | P | 111,705 | 34 | *ccsA* | SSC | 111,705 | 34 | *ccsA* | SSC |
| 38 | P | 112,126 | 30 | *ccsA-ndhD* | IGS | 112,169 | 30 | *ccsA-ndhD* | IGS |
| 39 | R | 112,251 | 31 | *ccsA-ndhD* | IGS | 112,251 | 31 | *ccsA-ndhD* | IGS |
| 40 | P | 141,225 | 35 | *trnL-CAA-ycf15* |  | 141,225 | 35 | *trnL-CAA-ycf15* | IGS |
| 41 | F | 143,797 | 37 | *ycf2* | IRa | 143,845 | 37 | *ycf2* | IRa |
| 42 | F | 143,801 | 57 | *ycf2* | IRa | 143,825 | 57 | *ycf2* | IRa |
| 43 | F | 143,821 | 37 | *ycf2* | IRa | 143,845 | 37 | *ycf2* | IRa |
| 44 | F | 143,830 | 31 | *ycf2* | IRa | 143,854 | 31 | *ycf2* | IRa |
| 45 | F | 146,174 | 30 | *ycf2* | IRa | 146,195 | 30 | *ycf2* | IRa |
| *Fritillaria taipaiensis* | | | | | | | | | |
| 1 | P | 5,363 | 34 | *rps16* | intron | 5,363 | 34 | *rps16* | intron |
| 2 | F | 7,026 | 31 | *trnS-GCU* | LSC | 33,087 | 31 | *psbC-trnS-UGA* | IGS |
| 3 | P | 7,028 | 31 | *trnS-GCU* | LSC | 42,438 | 31 | *trnS-GGA* | LSC |
| 4 | F | 8,580 | 30 | *trnG-GCC* | LSC | 33,889 | 30 | *trnG-UCC* | LSC |
| 5 | P | 26,822 | 38 | *trnC-GCA-petN* | IGS | 26,822 | 38 | *trnC-GCA-petN* | LSC |
| 6 | P | 27,046 | 65 | *petN-psbM* | IGS | 27,046 | 65 | *petN-psbM* | LSC |
| 7 | F | 29,999 | 38 | *trnT-GGU-psbD* | IGS | 30,012 | 38 | *trnT-GGU-psbD* | LSC |
| 8 | P | 33,086 | 32 | *psbC-trnS-UGA* | IGS | 42,440 | 32 | *trnS-GGA* | LSC |
| 9 | F | 36,109 | 50 | *psaB* | LSC | 38,333 | 50 | *psaA* | LSC |
| 10 | F | 36,132 | 30 | *psaB* | LSC | 38,356 | 30 | *psaA* | LSC |
| 11 | F | 40,992 | 39 | *ycf3* | intron | 96,032 | 39 | *rps12-trnV-GAC* | IGS |
| 12 | P | 40,992 | 39 | *ycf3* | intron | 137,087 | 39 | *trnV-GAC-rps12* | IGS |
| 13 | C | 43,857 | 31 | *trnT-UGU-trnL-UAA* | IGS | 64,506 | 31 | *psaJ-rpl33* | IGS |
| 14 | P | 43,979 | 33 | *trnT-UGU-trnL-UAA* | IGS | 43,979 | 33 | *trnT-UGU-trnL-UAA* | IGS |
| 15 | P | 44,081 | 32 | *trnT-UGU-trnL-UAA* | IGS | 44,081 | 32 | *trnT-UGU-trnL-UAA* | IGS |
| 16 | R | 77,798 | 31 | *rps8-rpl14* | IGS | 77,798 | 31 | *rps8-rpl14* | IGS |
| 17 | P | 81,117 | 32 | *rpl22-rps19* | IGS | 81,120 | 32 | *rpl22-rps19* | IGS |
| 18 | R | 81,172 | 32 | *rpl22-rps20* | IGS | 111,059 | 32 | *rpl32-trnL-UAG* | IGS |
| 19 | F | 86,289 | 30 | *ycf2* | IRb | 86,310 | 30 | *ycf2* | IRb |
| 20 | P | 86,289 | 30 | *ycf2* | IRb | 146,818 | 30 | *ycf2* | IRa |
| 21 | P | 86,310 | 30 | *ycf2* | IRb | 146,839 | 30 | *ycf2* | IRa |
| 22 | F | 88,629 | 57 | *ycf2* | IRb | 88,653 | 57 | *ycf2* | IRb |
| 23 | P | 88,629 | 57 | *ycf2* | IRb | 144,448 | 57 | *ycf2* | IRa |
| 24 | F | 88,629 | 37 | *ycf2* | IRb | 88,677 | 37 | *ycf2* | IRb |
| 25 | P | 88,629 | 37 | *ycf2* | IRb | 144,444 | 37 | *ycf2* | IRa |
| 26 | P | 88,653 | 57 | *ycf2* | IRb | 144,472 | 57 | *ycf2* | IRa |
| 27 | F | 88,658 | 32 | *ycf2* | IRb | 88,682 | 32 | *ycf2* | IRb |
| 28 | P | 88,658 | 32 | *ycf2* | IRb | 144,444 | 32 | *ycf2* | IRa |
| 29 | P | 88,677 | 37 | *ycf2* | IRb | 144,492 | 37 | *ycf2* | IRa |
| 30 | P | 88,682 | 32 | *ycf2* | IRb | 144,468 | 32 | *ycf2* | IRa |
| 31 | P | 91,511 | 35 | *trnL-CAA* | IRb | 91,511 | 35 | *trnL-CAA* | IRb |
| 32 | F | 91,511 | 35 | *trnL-CAA* | IRb | 141,612 | 35 | *trnL-CAA-ycf15* | IRa |
| 33 | P | 112,085 | 34 | *ccsA* | SSC | 112,085 | 34 | *ccsA* | SSC |
| 34 | P | 112,506 | 30 | *ccsA-ndhD* | IGS | 112,549 | 30 | *ccsA-ndhD* | IGS |
| 35 | R | 112,631 | 31 | *ccsA-ndhD* | IGS | 112,631 | 31 | *ccsA-ndhD* | IGS |
| 36 | P | 141,612 | 35 | *trnL-CAA-ycf15* | IGS | 141,612 | 35 | *trnL-CAA-ycf15* | IGS |
| 37 | F | 144,444 | 37 | *ycf2* | IRa | 144,492 | 37 | *ycf2* | IRa |
| 38 | F | 144,448 | 57 | *ycf2* | IRa | 144,472 | 57 | *ycf2* | IRa |
| 39 | F | 144,468 | 37 | *ycf2* | IRa | 144,492 | 37 | *ycf2* | IRa |
| 40 | F | 144,477 | 31 | *ycf2* | IRa | 144,501 | 31 | *ycf2* | IRa |
| 41 | F | 146,821 | 30 | *ycf2* | IRa | 146,842 | 30 | *ycf2* | IRa |
| *Fritillaria yuzhongensis* | | | | | | | | | |
| 1 | P | 1 | 95 | *trnH-psbA* | IGS | 81,512 | 95 | *rps19* | LSC |
| 2 | C | 4,092 | 31 | *trnK-UUU-rps16* | IGS | 45,322 | 31 | *trnL-UAA-trnF-GAA* | IGS |
| 3 | P | 5,465 | 34 | *rps16* | intron | 5,465 | 34 | *rps16* | intron |
| 4 | F | 7,128 | 31 | *psbI-trnS-GCU* | IGS | 33,185 | 31 | *trnS-UGA* | LSC |
| 5 | P | 7,130 | 31 | *psbI-trnS-GCU* | IGS | 42,537 | 31 | *trnS-GGA* | LSC |
| 6 | F | 8,661 | 30 | *trnG-GCC* | LSC | 33,987 | 30 | *psbZ-trnG-UCC* | IGS |
| 7 | P | 26,909 | 38 | *trnC-GCA-petN* | IGS | 26,909 | 38 | *trnC-GCA-petN* | IGS |
| 8 | P | 27,133 | 65 | *petN-psbM* | IGS | 27,133 | 65 | *petN-psbM* | IGS |
| 9 | F | 28,240 | 30 | *psbM-trnD-GUC* | IGS | 62,574 | 30 | *psbE-petL* | IGS |
| 10 | F | 28,613 | 31 | *trnD-GUC-trnY-GUA* | IGS | 28,636 | 31 | *trnD-GUC-trnY-GUA* | IGS |
| 11 | F | 30,108 | 38 | *trnT-GGU-psbD* | IGS | 30,121 | 38 | *trnT-GGU-psbD* | IGS |
| 12 | P | 33,184 | 32 | *psbC-trnS-UGA* | IGS | 42,539 | 32 | *trnS-GGA* | LSC |
| 13 | F | 36,207 | 50 | *psaB* | LSC | 38,431 | 50 | *psaA* | LSC |
| 14 | F | 36,230 | 30 | *psaB* | LSC | 38,454 | 30 | *psaA* | LSC |
| 15 | F | 41,090 | 39 | *ycf3* | intron | 96,092 | 39 | *rps12-trnV-GAC* | IGS |
| 16 | P | 41,090 | 39 | *ycf3* | intron | 137,121 | 39 | *trnV-GAC-rps12* | IGS |
| 17 | C | 43,908 | 31 | *trnT-UGU-trnL-UAA* | IGS | 64,568 | 31 | *psaJ-rpl33* | IGS |
| 18 | P | 44,031 | 33 | *trnT-UGU-trnL-UAA* | IGS | 44,031 | 33 | *rps4-trnT-UGU* | IGS |
| 19 | P | 44,133 | 32 | *trnT-UGU-trnL-UAA* | IGS | 44,133 | 32 | *rps4-trnT-UGU* | IGS |
| 20 | R | 44,302 | 30 | *trnT-UGU-trnL-UAA* | IGS | 62,580 | 30 | *psbE-petL* | IGS |
| 21 | F | 44,305 | 31 | *trnT-UGU-trnL-UAA* | IGS | 62,578 | 31 | *psbE-petL* | IGS |
| 22 | P | 44,307 | 31 | *trnT-UGU-trnL-UAA* | IGS | 45,317 | 31 | *trnL-UAA-trnF-GAA* | IGS |
| 23 | R | 44,307 | 30 | *trnT-UGU-trnL-UAA* | IGS | 81,162 | 30 | *rpl22-rps19* | IGS |
| 24 | R | 45,325 | 32 | *trnL-UAA-trnF-GAA* | IGS | 45,325 | 32 | *trnL-UAA-trnF-GAA* | IGS |
| 25 | F | 62,576 | 30 | *psbE-petL* | IGS | 62,577 | 30 | *psbE-petL* | IGS |
| 26 | R | 77,851 | 31 | *rps8-rpl14* | IGS | 77,851 | 31 | *rps8-rpl14* | IGS |
| 27 | R | 81,166 | 35 | *rpl22-rps19* | IGS | 81,166 | 35 | *rpl22-rps19* | IGS |
| 28 | R | 81,233 | 30 | *rpl22-rps19* | IGS | 111,120 | 30 | *rpl32-trnL-UAG* | IGS |
| 29 | F | 86,349 | 30 | *ycf2* | IRb | 86,370 | 30 | *ycf2* | IRb |
| 30 | P | 86,349 | 30 | *ycf2* | IRb | 146,852 | 30 | *ycf2* | IRa |
| 31 | F | 88,689 | 57 | *ycf2* | IRb | 88,713 | 57 | *ycf2* | IRb |
| 32 | P | 88,689 | 57 | *ycf2* | IRb | 144,482 | 57 | *ycf2* | IRa |
| 33 | F | 88,689 | 37 | *ycf2* | IRb | 88,737 | 37 | *ycf2* | IRb |
| 34 | P | 88,689 | 37 | *ycf2* | IRb | 144,478 | 37 | *ycf2* | IRa |
| 35 | P | 88,713 | 57 | *ycf2* | IRb | 144,506 | 57 | *ycf2* | IRa |
| 36 | F | 88,718 | 32 | *ycf2* | IRb | 88,742 | 32 | *ycf2* | IRb |
| 37 | P | 88,718 | 32 | *ycf2* | IRb | 144,478 | 32 | *ycf2* | IRa |
| 38 | P | 88,737 | 37 | *ycf2* | IRb | 144,526 | 37 | *ycf2* | IRa |
| 39 | P | 88,742 | 32 | *ycf2* | IRb | 144,502 | 32 | *ycf2* | IRa |
| 40 | P | 91,571 | 35 | *ycf15-trnL-CAA* | IGS | 91,571 | 35 | *ycf15-trnL-CAA* | IGS |
| 41 | F | 91,571 | 35 | *ycf15-trnL-CAA* | IGS | 141,646 | 35 | *trnL-CAA-ycf15* | IGS |
| 42 | P | 112,144 | 34 | *ccsA* | SSC | 112,144 | 34 | *ccsA* | SSC |
| 43 | P | 112,565 | 30 | *ccsA-ndhD* | IGS | 112,608 | 30 | *ccsA-ndhD* | IGS |
| 44 | R | 112,690 | 31 | *ccsA-ndhD* | IGS | 112,690 | 31 | *ccsA-ndhD* | IGS |
| 45 | P | 141,646 | 35 | *trnL-CAA-ycf15* | IGS | 141,646 | 35 | *trnL-CAA-ycf15* | IGS |
| 46 | F | 144,478 | 37 | *ycf2* | IRa | 144,526 | 37 | *ycf2* | IRa |
| 47 | F | 144,482 | 57 | *ycf2* | IRa | 144,506 | 57 | *ycf2* | IRa |
| 48 | F | 144,502 | 37 | *ycf2* | IRa | 144,526 | 37 | *ycf2* | IRa |
| 49 | F | 144,511 | 31 | *ycf2* | IRa | 144,535 | 31 | *ycf2* | IRa |
| *Fritillaria sinica* | | | | | | | | | |
| 1 | P | 1 | 95 | *trnH-psbA* | IGS | 81,922 | 95 | *rps19-trnH-GUG* | IGS |
| 2 | P | 5,433 | 34 | *rps16* | intron | 5,433 | 34 | *rps16* | intron |
| 3 | F | 7,106 | 31 | *psbI* | LSC | 33,379 | 31 | *psbC-trnS-UGA* | IGS |
| 4 | P | 7,108 | 31 | *psbI* | LSC | 42,732 | 31 | *trnS-GGA* | LSC |
| 5 | F | 8,642 | 30 | *trnG-GCC* | LSC | 34,179 | 30 | *trnG-UCC* | LSC |
| 6 | P | 26,919 | 38 | *trnC-GCA-petN* | IGS | 26,919 | 38 | *trnC-GCA-petN* | IGS |
| 7 | P | 27,143 | 65 | *petN-psbM* | IGS | 27,143 | 65 | *petN-psbM* | IGS |
| 8 | F | 28,258 | 32 | *psbM-trnD-GUC* | IGS | 81,639 | 32 | *rpl22-rps19* | IGS |
| 9 | F | 28,264 | 30 | *psbM-trnD-GUC* | IGS | 28,266 | 30 | *psbM-trnD-GUC* | IGS |
| 10 | F | 28,265 | 31 | *psbM-trnD-GUC* | IGS | 44,739 | 31 | *trnT-UGU-trnL-UAA* | IGS |
| 11 | R | 28,267 | 31 | *psbM-trnD-GUC* | IGS | 28,267 | 31 | *psbM-trnD-GUC* | IGS |
| 12 | F | 28,268 | 30 | *psbM-trnD-GUC* | IGS | 28,269 | 30 | *psbM-trnD-GUC* | IGS |
| 13 | F | 28,269 | 31 | *psbM-trnD-GUC* | IGS | 81,647 | 31 | *rpl22-rps19* | IGS |
| 14 | R | 28,269 | 30 | *psbM-trnD-GUC* | IGS | 44,740 | 30 | *trnT-UGU-trnL-UAA* | IGS |
| 15 | F | 28,270 | 30 | *psbM-trnD-GUC* | IGS | 44,539 | 30 | *trnT-UGU-trnL-UAA* | IGS |
| 16 | R | 28,271 | 30 | *psbM-trnD-GUC* | IGS | 44,532 | 30 | *trnT-UGU-trnL-UAA* | IGS |
| 17 | F | 30,290 | 38 | *trnT-GGU-psbD* | IGS | 30,303 | 38 | *trnT-GGU-psbD* | IGS |
| 18 | P | 33,378 | 32 | *psbC-trnS-UGA* | IGS | 42,734 | 32 | *trnS-GGA* | IGS |
| 19 | F | 36,398 | 50 | *psaB* | LSC | 38,622 | 50 | *psaA* | LSC |
| 20 | F | 36,416 | 32 | *psaB* | LSC | 38,640 | 32 | *psaA* | LSC |
| 21 | F | 41,287 | 39 | *ycf3* | intron | 96,501 | 39 | *rps12-trnV-GAC* | IGS |
| 22 | P | 41,287 | 39 | *ycf3* | intron | 137,541 | 39 | *trnV-GAC-rps12* | IGS |
| 23 | C | 44,156 | 31 | *trnT-UGU-trnL-UAA* | IGS | 64,975 | 31 | *psaJ-rpl33* | IGS |
| 24 | P | 44,278 | 33 | *trnT-UGU-trnL-UAA* | IGS | 44,278 | 33 | *trnT-UGU-trnL-UAA* | IGS |
| 25 | P | 44,381 | 30 | *trnT-UGU-trnL-UAA* | IGS | 44,383 | 30 | *trnT-UGU-trnL-UAA* | IGS |
| 26 | R | 44,531 | 31 | *trnT-UGU-trnL-UAA* | IGS | 44,747 | 31 | *trnT-UGU-trnL-UAA* | IGS |
| 27 | R | 44,540 | 31 | *trnT-UGU-trnL-UAA* | IGS | 44,737 | 31 | *trnT-UGU-trnL-UAA* | IGS |
| 28 | R | 44,742 | 30 | *trnT-UGU-trnL-UAA* | IGS | 81,645 | 30 | *rpl22-rps19* | IGS |
| 29 | F | 58,581 | 30 | *ycf4-cemA* | IGS | 58,610 | 30 | *ycf4-cemA* | IGS |
| 30 | R | 78,261 | 31 | *rps8-rpl14* | IGS | 78,261 | 31 | *rps8-rpl14* | IGS |
| 31 | F | 89,099 | 57 | *ycf2* | IRb | 89,123 | 57 | *ycf2* | IRb |
| 32 | P | 89,099 | 57 | *ycf2* | IRb | 144,901 | 57 | *ycf2* | IRa |
| 33 | F | 89,099 | 37 | *ycf2* | IRb | 89,147 | 37 | *ycf2* | IRb |
| 34 | P | 89,099 | 37 | *ycf2* | IRb | 144,897 | 37 | *ycf2* | IRa |
| 35 | P | 89,123 | 57 | *ycf2* | IRb | 144,925 | 57 | *ycf2* | IRa |
| 36 | F | 89,128 | 32 | *ycf2* | IRb | 89,152 | 32 | *ycf2* | IRb |
| 37 | P | 89,128 | 32 | *ycf2* | IRb | 144,897 | 32 | *ycf2* | IRa |
| 38 | P | 89,147 | 37 | *ycf2* | IRb | 144,945 | 37 | *ycf2* | IRa |
| 39 | P | 89,152 | 32 | *ycf2* | IRb | 144,921 | 32 | *ycf2* | IRa |
| 40 | P | 91,981 | 35 | *ycf15-trnL-CAA* | IGS | 91,981 | 35 | *ycf15-trnL-CAA* | IGS |
| 41 | F | 91,981 | 35 | *ycf16-trnL-CAA* | IGS | 142,065 | 35 | *trnL-CAA-ycf2* | IGS |
| 42 | P | 112,544 | 34 | *ccsA* | SSC | 112,544 | 34 | *ccsA* | SSC |
| 43 | P | 112,965 | 30 | *ccsA-ndhD* | IGS | 113,008 | 30 | *ccsA-ndhD* | IGS |
| 44 | R | 113,090 | 31 | *ccsA-ndhD* | IGS | 113,090 | 31 | *ccsA-ndhD* | IGS |
| 45 | P | 142,065 | 35 | *ycf2* | IRa | 142,065 | 35 | *ycf2* | IRa |
| 46 | F | 144,897 | 37 | *ycf2* | IRa | 144,945 | 37 | *ycf2* | IRa |
| 47 | F | 144,901 | 57 | *ycf2* | IRa | 144,925 | 57 | *ycf2* | IRa |
| 48 | F | 144,921 | 37 | *ycf2* | IRa | 144,945 | 37 | *ycf2* | IRa |
| 49 | F | 144,930 | 31 | *ycf2* | IRa | 144,954 | 31 | *ycf2* | IRa |
| *Fritillaria dajinensis* | | | | | | | | | |
| 1 | P | 5,338 | 34 | *rps16* | intron | 5,338 | 34 | *rps16* | intron |
| 2 | F | 7,010 | 31 | *psbI-trnS-GCU* | IGS | 33,286 | 31 | *psbC-trnS-UGA* | IGS |
| 3 | P | 7,012 | 31 | *psbI-trnS-GCU* | IGS | 42,635 | 31 | *trnS-GGA* | LSC |
| 4 | F | 8,550 | 30 | *trnG-GCC* | LSC | 34,086 | 30 | *trnG-UCC* | LSC |
| 5 | P | 26,831 | 38 | *trnC-GCA-petN* | IGS | 26,831 | 38 | *trnC-GCA-petN* | IGS |
| 6 | P | 27,055 | 65 | *petN-psbM* | IGS | 27,055 | 65 | *petN-psbM* | IGS |
| 7 | F | 28,170 | 31 | *psbM-trnD-GUC* | IGS | 81,538 | 31 | *rps19* | LSC |
| 8 | P | 28,173 | 30 | *psbM-trnD-GUC* | IGS | 45,461 | 30 | *trnL-UAA-trnF-GAA* | IGS |
| 9 | P | 28,177 | 31 | *psbM-trnD-GUC* | IGS | 45,457 | 31 | *trnL-UAA-trnF-GAA* | IGS |
| 10 | F | 28,180 | 30 | *psbM-trnD-GUC* | IGS | 81,546 | 30 | *rps19* | LSC |
| 11 | C | 28,181 | 30 | *psbM-trnD-GUC* | IGS | 45,461 | 30 | *trnL-UAA-trnF-GAA* | LSC |
| 12 | F | 30,197 | 38 | *trnT-GGU-psbD* | IGS | 30,210 | 38 | *trnT-GGU-psbD* | IGS |
| 13 | P | 33,285 | 32 | *psbC-trnS-UGA* | IGS | 42,637 | 32 | *ycf3* | intron |
| 14 | F | 36,305 | 50 | *psaB* | LSC | 38,529 | 50 | *psaA* | LSC |
| 15 | F | 36,323 | 32 | *psaB* | LSC | 38,547 | 32 | *psaA* | LSC |
| 16 | F | 41,193 | 39 | *ycf3* | intron | 96,399 | 39 | *rps12-trnV-GAC* | IRa |
| 17 | P | 41,193 | 39 | *ycf3* | intron | 137,444 | 39 | *trnV-GAC-rps12* | IGS |
| 18 | C | 44,059 | 31 | *trnT-UGU-trnL-UAA* | LSC | 64,874 | 31 | *psaJ-rpl33* | IGS |
| 19 | P | 44,182 | 33 | *trnT-UGU-trnL-UAA* | LSC | 44,182 | 33 | *trnT-UGU-trnL-UAA* | LSC |
| 20 | R | 45,455 | 35 | *trnL-UAA-trnF-GAA* | IGS | 45,455 | 35 | *trnL-UAA-trnF-GAA* | IGS |
| 21 | P | 45,466 | 30 | *trnL-UAA-trnF-GAA* | IGS | 81,539 | 30 | *rps19* | LSC |
| 22 | F | 58,478 | 30 | *ycf4-cemA* | IGS | 58,507 | 30 | *ycf4-cemA* | IGS |
| 23 | R | 78,158 | 31 | *rps8-rpl14* | IGS | 78,158 | 31 | *rps8-rpl14* | IGS |
| 24 | F | 86,657 | 30 | *ycf2* | IRb | 86,678 | 30 | *ycf2* | IRb |
| 25 | P | 86,657 | 30 | *ycf2* | IRb | 147,174 | 30 | *ycf2* | IRa |
| 26 | P | 86,678 | 30 | *ycf2* | IRb | 147,195 | 30 | *ycf2* | IRa |
| 27 | F | 88,997 | 57 | *ycf2* | IRb | 89,021 | 57 | *ycf2* | IRb |
| 28 | P | 88,997 | 57 | *ycf2* | IRb | 144,804 | 57 | *ycf2* | IRa |
| 29 | F | 88,997 | 37 | *ycf2* | IRb | 89,045 | 37 | *ycf2* | IRb |
| 30 | P | 88,997 | 37 | *ycf2* | IRb | 144,800 | 37 | *ycf2* | IRa |
| 31 | P | 89,021 | 57 | *ycf2* | IRb | 144,828 | 57 | *ycf2* | IRa |
| 32 | F | 89,026 | 32 | *ycf2* | IRb | 89,050 | 32 | *ycf2* | IRb |
| 33 | P | 89,026 | 32 | *ycf2* | IRb | 144,800 | 32 | *ycf2* | IRa |
| 34 | P | 89,045 | 37 | *ycf2* | IRb | 144,848 | 37 | *ycf2* | IRa |
| 35 | P | 89,050 | 32 | *ycf2* | IRb | 144,824 | 32 | *ycf2* | IRa |
| 36 | P | 91,879 | 35 | *trnL-CAA-ndhB* | IGS | 91,879 | 35 | *trnL-CAA-ndhB* | IGS |
| 37 | F | 91,879 | 35 | *trnL-CAA-ndhB* | IGS | 141,968 | 35 | *trnL-CAA-ycf15* | IGS |
| 38 | P | 112,442 | 34 | *ccsA* | LSC | 112,442 | 34 | *ccsA* | LSC |
| 39 | P | 112,863 | 30 | *ccsA-ndhD* | IGS | 112,906 | 30 | *ccsA* | LSC |
| 40 | R | 112,988 | 31 | *ccsA-ndhD* | IGS | 112,988 | 31 | *ccsA-ndhD* | IGS |
| 41 | P | 141,968 | 35 | *ccsA-ndhD* | IGS | 141,968 | 35 | *trnL-CAA-ycf15* | IGS |
| 42 | F | 144,800 | 37 | *ycf2* | IRa | 144,848 | 37 | *ycf2* | IRa |
| 43 | F | 144,804 | 57 | *ycf2* | IRa | 144,828 | 57 | *ycf2* | IRa |
| 44 | F | 144,824 | 37 | *ycf2* | IRa | 144,848 | 37 | *ycf2* | IRa |
| 45 | F | 144,833 | 31 | *ycf2* | IRa | 144,857 | 31 | *ycf2* | IRa |
| 46 | F | 147,177 | 30 | *ycf2* | IRa | 147,198 | 30 | *ycf2* | IRa |
