# Supplementary material for: Phylogenetic analysis of Fritillaria cirrhosa D. Don and its closely related species based on complete chloroplast genomes
Source: PeerJ. 2019 Aug 21;7:e7480. doi: 10.7717/peerj.7480 (PMC6708372; doi:10.7717/peerj.7480)
Supplement: Table S6 [file peerj-07-7480-s008.docx]

Table S6. Frequency of complement repeats by length in eight *Fritillaria*

|  | 30-44 | 45-59 | 60-74 | 75-89 | >90 |
| --- | --- | --- | --- | --- | --- |
| *F. cirrhosa* | 1 | 0 | 0 | 0 | 0 |
| *F. sichuanica* | 1 | 0 | 0 | 0 | 0 |
| *F. przewalskii* | 1 | 0 | 0 | 0 | 0 |
| *F. unibracteata* | 3 | 0 | 0 | 0 | 0 |
| *F. taipaiensis* | 1 | 0 | 0 | 0 | 0 |
| *F. yuzhongensis* | 2 | 0 | 0 | 0 | 0 |
| *F. sinica* | 1 | 0 | 0 | 0 | 0 |
| *F. dajinensis* | 2 | 0 | 0 | 0 | 0 |
