# Supplementary material for: Phylogenetic analysis of Fritillaria cirrhosa D. Don and its closely related species based on complete chloroplast genomes
Source: PeerJ. 2019 Aug 21;7:e7480. doi: 10.7717/peerj.7480 (PMC6708372; doi:10.7717/peerj.7480)
Supplement: Table S7 [file peerj-07-7480-s009.docx]

Table S7. Regions of highly variable sequences of *Fritillaria*

| NO. | High variable region | Length | Variable sites | parsimony information sites | Nucleotide diversity |
| --- | --- | --- | --- | --- | --- |
| 1 | *trnS-GCU-trnR-UCU* | 1,666 | 16 | 9 | 0.00393 |
| 2 | *rpoB-psbD* | 5,025 | 69 | 38 | 0.00468 |
| 3 | *rps4-trnF-GAA* | 2,042 | 36 | 13 | 0.00586 |
| 4 | *petA-psbL* | 2,761 | 20 | 10 | 0.00608 |
| 5 | *ndhF-ndhD* | 2,625 | 41 | 16 | 0.00543 |
| 6 | *ycf1* | 5,550 | 75 | 30 | 0.00474 |
