# Supplementary material for: Phylogenetic analysis of Fritillaria cirrhosa D. Don and its closely related species based on complete chloroplast genomes
Source: PeerJ. 2019 Aug 21;7:e7480. doi: 10.7717/peerj.7480 (PMC6708372; doi:10.7717/peerj.7480)
Supplement: Table S8 [file peerj-07-7480-s010.docx]

Table S8. Length of each dataset matrix used for phylogeny construction and the best fitting models tested by jModeltest v.2.1.7 based on Akaike Information Criterion.

| Dataset | Length (bp) | Best substitution models |
| --- | --- | --- |
| Chloroplast genome  (Only contained one IR) | 127,964 | GTR+I |
|  |  |  |
| Lange single copy (LSC) | 83,741 | GTR+I+G |
| Small single copy (SSC) | 17,708 | GTR+I+G |
| Inverted repeats (IR) | 26,302 | GTR+I |
| Protein coding genes (CDS) | 68,613 | GTR+I |
